# Macrophage membrane‒biomimetic nanoparticles target inflammatory microenvironment for epilepsy treatment

**DOI:** 10.7150/thno.99260

**Published:** 2024-10-07

**Authors:** Chao Geng, Xinghui Ren, Peipei Cao, Xiaoqi Chu, Penghu Wei, Quanlei Liu, Yongchang Lu, Bin Fu, Wenyou Li, Yuhao Li, Guoguang Zhao

**Affiliations:** 1 Optometry Institute, School of Medicine Nankai University, Tianjin 300071, China.; 2 Department of Neurosurgery, Xuanwu Hospital Capital Medical University, Beijing Municipal Geriatric Medical Research Center, Beijing 100053, China.; 3 College of Chemistry, Research Center for Analytical Sciences, State Key Laboratory of Medicinal Chemical Biology, Tianjin Key Laboratory of Biosensing and Molecular Recognition, Nankai University, Tianjin 300071, China.; 4 Central Laboratory, Xuanwu Hospital Capital Medical University, Beijing Municipal Geriatric Medical Research Center, Beijing 100053, China.; 5 Department of Pathology, School of Medicine Nankai University, Tianjin 300071, China.; 6 National Medical Center for Neurological Diseases, Beijing 100053, China.; 7 Clinical Research Center for Epilepsy Capital Medical University, Beijing 100053, China.

**Keywords:** epilepsy, death-related protein kinase 1, macrophage membrane biomimetic nanoparticles, inflammation, drug delivery

## Abstract

**Rationale:** The clinical treatment of epilepsy is faced with challenges. On the one hand, the effectiveness of existing antiepileptic drugs (AEDs) is limited by the blood‒brain barrier (BBB); on the other hand, changes in the inflammatory microenvironment during epileptogenesis are often neglected.

**Methods:** The death-associated protein kinase 1 inhibitor TC-DAPK6 and the fluorescent probe rhodamine B were encapsulated in hollow mesoporous silica nanocarriers (HMSNs), which were then coated with a macrophage membrane to prepare macrophage membrane-biomimetic nanoparticles, namely, MA@RT-HMSNs. *In vitro* biotoxicity, cellular uptake, BBB permeability and inflammatory targeting ability were evaluated in cells. The effects of MA@RT-HMSN treatment were explored by immunohistochemistry, TUNEL assay, Western blot analysis, quantitative real-time polymerase chain reaction, electroencephalogram recording and behavioural tests in kainic acid-induced acute and chronic epilepsy model mice.

**Results:** MA@RT-HMSNs showed excellent biocompatibility both *in vitro* and *in vivo*. MA@RT-HMSNs successfully crossed the BBB and exhibited increased efficacy in targeted delivery of TC-DAPK6 to inflammatory lesions in epileptic foci. Macrophage membrane coating conferred MA@RT-HMSNs with higher stability, greater cellular uptake, and enhanced TC-DAPK6 bioavailability. Furthermore, MA@RT-HMSNs exerted beneficial therapeutic effects on acute and chronic epilepsy models by alleviating microenvironment inflammation, preventing neuronal death, and inhibiting neuronal excitability and gliosis.

**Conclusions:** MA@RT-HMSNs target inflammatory foci to inhibit death-related protein kinase 1 and exert antiepileptic effects. This study provides a promising biomimetic nanodelivery system for targeted epilepsy therapy.

## Introduction

Epilepsy is one of the most common neurological disorders and affects approximately 70 million people worldwide, making it a global public health issue 1. Epilepsy is characterized by unprovoked and recurrent seizures and brain dysfunction triggered by aberrant neuronal electrical activity 2. Antiepileptic drugs (AEDs) constitute the mainstay of clinical treatment for most patients with epilepsy 3. Although more than 20 kinds of AEDs are currently in use, seizures cannot be effectively controlled in approximately one-third of patients due to drug resistance, and these patients can even develop refractory epilepsy, which is life-threatening 4. Systemic administration of AEDs is associated with severe side effects. More importantly, the blood‒brain barrier (BBB), a semipermeable membrane that restricts the passage of molecules into the brain, not only protects the central nervous system (CNS) from potentially harmful molecules in the blood but also limits drug delivery to the brain 5. The inadequate penetration of AEDs across the BBB inhibits the delivery of the required drug concentrations to epileptic lesions to maintain the therapeutic window 6, 7. Therefore, there is an urgent need to explore new molecular targets and drug delivery systems that can effectively cross the BBB and target epileptic lesions.

Death-associated protein kinase 1 (DAPK1) is a calcium/calmodulin-dependent serine/threonine kinase that is abundant in the brain, especially in the hippocampus 8. DAPK1 is involved in mediating physiological or pathological processes such as apoptosis, autophagy, inflammation, oxidative stress, neurogenesis, neuronal damage, and synaptic transmission 9, 10. DAPK1 dysregulation has been detected in several neurological disorders, including Alzheimer's disease, cerebral ischaemia, epilepsy and depression 11-13. It was shown that the activity of DAPK1 was increased and its expression was upregulated in the hippocampus of epilepsy patients, which may contribute to neuronal damage by mediating cell death signalling pathways 14. In animal experiments, DAPK1 overexpression was detected in the hippocampus of epileptic mice, and subsequently, DAPK1 gene knockout or pharmacological inhibition of DAPK1 alleviated seizures in these mice 15, 16. Thus, DAPK1 has potential as a novel therapeutic target for epilepsy. Structure-based virtual screening and identification revealed that TC-DAPK6 is the most potent small molecule inhibitor of DAPK1 17, 18. However, the hydrophobicity of TC-DAPK6 limits its effective concentration and BBB permeability *in vivo*. In addition, the possible consequences of an increased drug dose causing systemic side effects need to be considered 19.

Nanoparticles have shown great potential as drug delivery carriers for the treatment of CNS diseases 20. The encapsulation of drugs into nanoparticles improves drug stability, prolongs blood circulation time, controls drug release and reduces side effects 21. Mesoporous silica nanoparticles (MSNs) are inorganic nanomaterials synthesized with silica as the precursor that feature an adjustable mesoporous structure, stable structural characteristics and suitable biocompatibility 22, 23. Compared with MSNs, hollow mesoporous silica nanoparticles (HMSNs) have a large hollow structure, large surface area and high drug loading capacity 24. Additionally, HMSNs can simultaneously carry therapeutic drugs, imaging agents and targeting ligands via functionalization 25. Fluorochrome rhodamine B (RhB), a common red fluorescent compound, was used as a probe because of its superior photophysical properties and biocompatibility, facilitating the precise tracking of nanoparticles 23, 26. Recently, cell membrane-coated nanoparticles, which are nanoparticle cores coated with cell membranes derived from specific cells, have attracted attention for their ability to mimic cellular functions. Cell membrane coatings allow nanoparticles to better adapt to complex physiological environments, present the antigenic properties of their original cells, increase their biocompatibility, and prolong their half-life 27. Macrophages, important phagocytes of the innate immune system, have the natural ability to respond to biological signals and to be recruited to the inflammatory area of the brain by adhering to endothelial cells and crossing the BBB 28. Owing to inflammation-directed chemotaxis, macrophage membrane-coated nanoparticles can promote the accumulation of drugs in inflammatory lesions affected by glioblastoma, acute ischaemic stroke and Alzheimer's disease 29. However, little is known about the application of macrophage membrane-biomimetic nanoparticles in epilepsy.

In this study, we focused on the modulation of DAPK1 in epilepsy. We aimed to inhibit DAPK1 targetedly and optimize the treatment route to overcome the deficiencies of the inhibitor TC-DAPK6, which is hydrophobic and has poor BBB permeability and a short half-life. Herein, TC-DAPK6 and RhB were loaded into the pores of HMSNs to synthesize RT-HMSNs. The macrophage membrane was coated on the surface of the RT-HMSNs to prepare novel macrophage membrane-biomimetic nanoparticles (MA@RT-HMSNs) that cross the BBB and target epileptic inflammatory lesions. The present work provides not only a feasible platform for precise inhibition of DAPK1 in inflammatory lesions of the brain but also a promising targeted therapy for epilepsy.

## Results

### Characterization of RT-HMSNs and MA@RT-HMSNs

**Figure 1:**
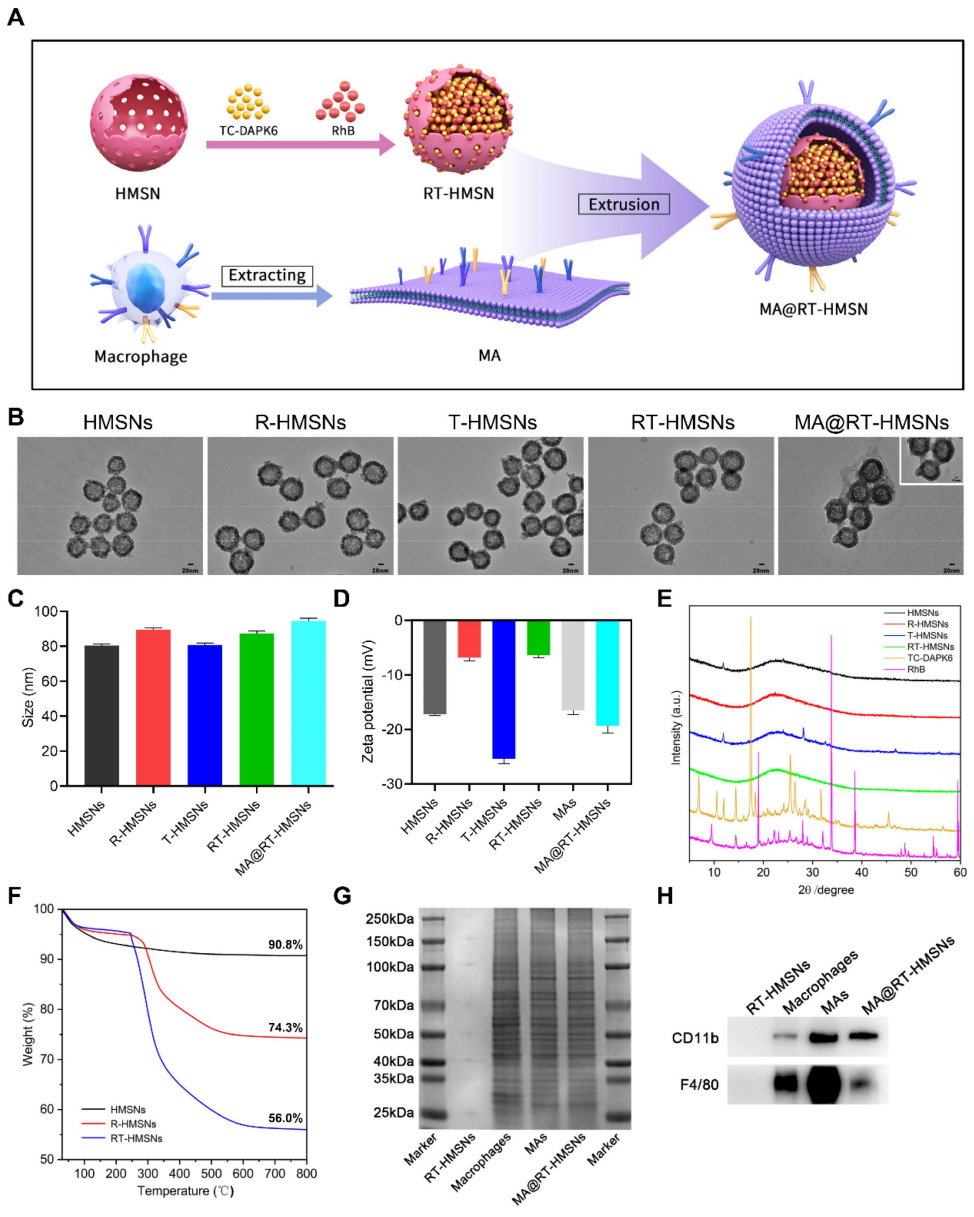
Preparation and characterization of RT-HMSNs and MA@RT-HMSNs. (A) Scheme for the synthesis of TC-DAPK6- and rhodamine B-loaded MA@RT-HMSNs. (B) TEM images of HMSNs, R-HMSNs, T-HMSNs, RT-HMSNs and MA@RT-HMSNs. (C) Statistical analysis of the particle size (ANOVA). (D) Zeta potentials of HMSNs, R-HMSNs, T-HMSNs, RT-HMSNs, MAs and MA@RT-HMSNs. (E) Powder X-ray diffraction (PXRD) analysis of HMSNs, R-HMSNs, T-HMSNs, RT-HMSNs, TC-DAPK6 and RhB. (F) TGA curves of HMSNs, R-HMSNs and RT-HMSNs. (G) Protein profiles and expression of (H) CD11b and F4/80 in RT-HMSNs, macrophages, MAs and MA@RT-HMSNs. Scale bar in (B), 20 nm; in the inset box, 10 nm.

The synthesis process consisted of three steps: first, TC-DAPK6 and RhB were loaded into HMSNs to obtain RT-HMSNs; second, the macrophage membrane was extracted; and finally, the macrophage membrane was wrapped onto the surface of RT-HMSNs by extrusion. A schematic illustration of the synthesis of MA@RT-HMSNs is shown in Figure 1A. DiO and 4′,6-diamidino-2-phenylindole (DAPI) staining of RAW264.7 cells revealed that the macrophage membranes were successfully isolated and fragmented after cell membrane extraction (Figure S1). Transmission electron microscopy (TEM) revealed that HMSNs, R-HMSNs, T-HMSNs, RT-HMSNs and MA@RT-HMSNs had uniform spherical morphologies and monodispersity (Figure 1B). The average particle sizes of HMSNs, R-HMSNs, T-HMSNs, and RT-HMSNs were 80, 89, 81 and 87 nm, respectively (Figure 1C). MA@RT-HMSNs were increased to an 8 nm-thick layer (Figure 1B, insert box and Figure 1C) of approximately 91 nm. Compared to that of the uncoated RT-HMSNs, the hydrodynamic diameter of the MA@RT-HMSNs increased from 122 to 190 nm, with a polydispersity index (PDI) of 0.457 (Figure S2). The zeta potentials of HMSNs, R-HMSNs, T-HMSNs and RT-HMSNs were -17.2, -6.84, -25.4 and -6.41 mV, respectively. However, the potential of the MA@RT-HMSNs was -19.3 mV, which was close to the surface potential of the macrophage membrane (MA) vesicles (-16.5 mV), further demonstrating that the cell membrane was successfully appended onto the surface of the RT-HMSNs (Figure 1D). The crystallographic structure was analysed via powder X-ray diffraction (PXRD). The HMSN was in a noncrystalline state with no characteristic diffraction peaks and only had wide diffuse peaks at 15° and 35°. TC-DAPK6 and RhB were both crystals with multiple strong diffraction peaks, whereas the diffraction peaks disappeared in the R-HMSNs, T-HMSNs and RT-HMSNs solid dispersion systems, indicating that they were highly dispersed in the HMSN material in an amorphous state (Figure 1E). Weight loss and thermal stability were characterized via thermogravimetric analysis (TGA). According to TGA, the initial weight loss occurred at approximately 250 °C, demonstrating the suitable thermal stability of the HMSN substrate. The final weight loss indicated that TC-DAPK6 and RhB were successfully loaded into the HMSNs at percentages of 18.3% and 16.5%, respectively (Figure 1F). RhB had a characteristic adsorption peak at 545 nm, and thus, the encapsulation efficiency of RhB was 55.9% based on the standard curves of absorbance and concentration detected by UV‒visible (UV‒vis) spectroscopy (Figure S3). The encapsulation efficiency of TC-DAPK6 in the RT-HMSNs was approximately 53.2%, as determined by high-performance liquid chromatography (HPLC). Moreover, SDS‒polyacrylamide gel electrophoresis (PAGE) analysis of the membrane proteins revealed that MA@RT-HMSNs retained most of the surficial cell membrane proteins and that mechanical extrusion did not alter the protein profile (Figure 1G). Western blot analysis revealed that the macrophage membrane markers CD11b and F4/80 were strongly expressed in macrophages, MAs and MA@RT-HMSNs, demonstrating that MA@RT-HMSNs retain the functions of macrophage membranes (Figure 1H). Overall, MA@RT-HMSNs were successfully prepared.

### *In vitro* and *in vivo* biotoxicity of MA@RT-HMSNs

**Figure 2:**
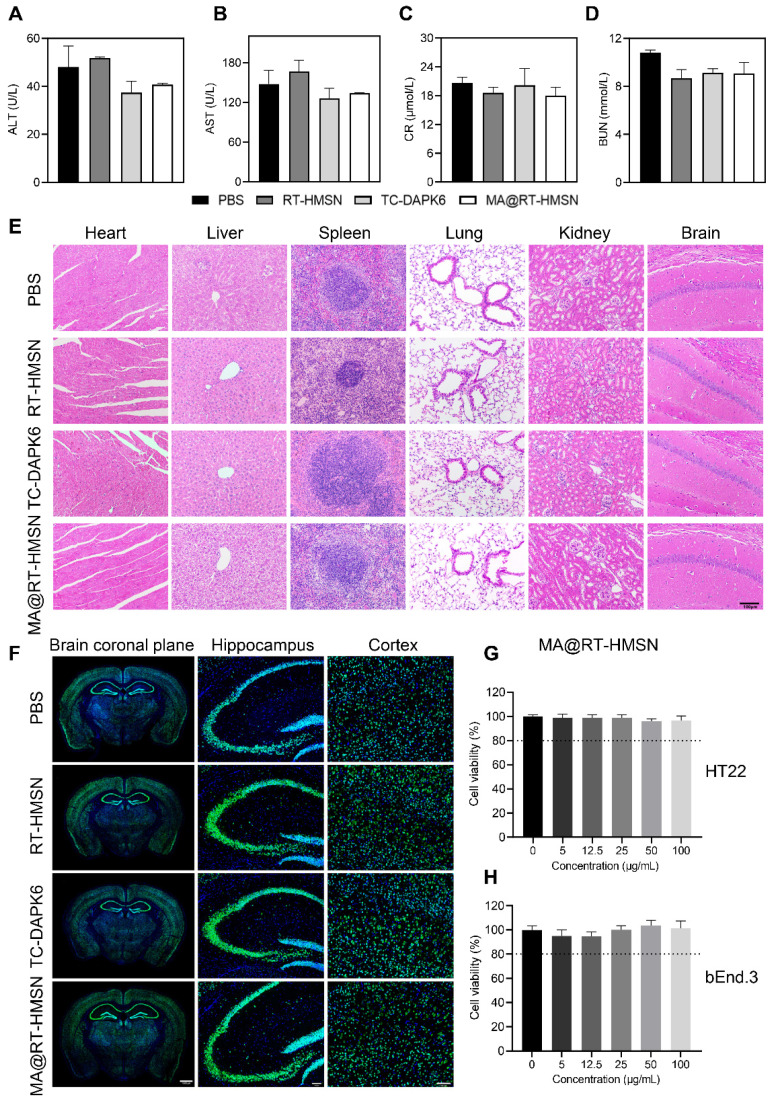
Biosafety evaluation of MA@RT-HMSNs. (A-D) Blood chemistry analysis of (A) ALT, (B) AST, (C) UR and (D) BUN in mice at 7 days post-PBS, RT-HMSN, TC-DAPK6 and MA@RT-HMSN injection (ANOVA; n = 4 in each group). (E) Representative images of H&E-stained major organs (heart, liver, spleen, lung, kidney and brain) in the four groups. (F) NeuN immunofluorescence staining of brain slices taken from the brain coronal plane, hippocampus and cortex in the four groups. (G and H) Cell viability of (G) HT22 and (H) bEnd.3 cells incubated with MA@RT-HMSNs at different concentrations (0, 5, 12.5, 25, 50, 100 μg/mL) for 24 h (ANOVA; n = 6 at each concentration). Scale bar in (E) and (F), 100 μm.

To assess *in vivo* toxicity, healthy mice were randomly divided into four groups: the PBS, RT-HMSN, TC-DAPK6 and MA@RT-HMSN groups. There was no significant difference in body weight before or after RT-HMSN, TC-DAPK6 or MA@RT-HMSN administration (Figure S4). Moreover, serum parameters, including alanine aminotransferase (ALT), aspartate aminotransferase (AST), creatinine (CR) and blood urea nitrogen (BUN), were within normal ranges in the RT-HMSN, TC-DAPK6 and MA@RT-HMSN groups compared with the PBS control group, demonstrating that no significant abnormalities in liver or kidney function were detected (Figure 2A-D). Haematoxylin and eosin (H&E) staining of the heart, liver, spleen, lung, kidney and brain revealed no obvious changes in tissue structure, cell loss, or inflammatory cell infiltration (Figure 2E). NeuN fluorescence staining of the brain was performed to evaluate neurotoxicity. The tissue structure in the coronal plane of the brain in the RT-HMSN, TC-DAPK6 and MA@RT-HMSN groups was similar to that in the PBS group, and no cell loss was observed in the magnified images of the hippocampus and cortex (Figure 2F). The *in vitro* experiments revealed that HT22 and bEnd.3 cells had high viability after incubation with MA@RT-HMSNs at concentrations ranging from 0 to 100 μg/mL for 24 h (Figure 2G and H). However, the viability of both cell lines decreased significantly after incubation with 100 μg/mL RT-HMSNs for 24 h (Figure S5A and B), indicating that the macrophage membrane coating further improved the biocompatibility of MA@RT-HMSNs compared with that of the uncoated RT-HMSNs. Collectively, these results demonstrate that MA@RT-HMSNs have low biotoxicity and excellent biocompatibility both *in vitro* and *in vivo*.

### Inflammation and apoptosis were reduced following MA@RT-HMSN treatment in a glutamate-induced cell injury model

**Figure 3:**
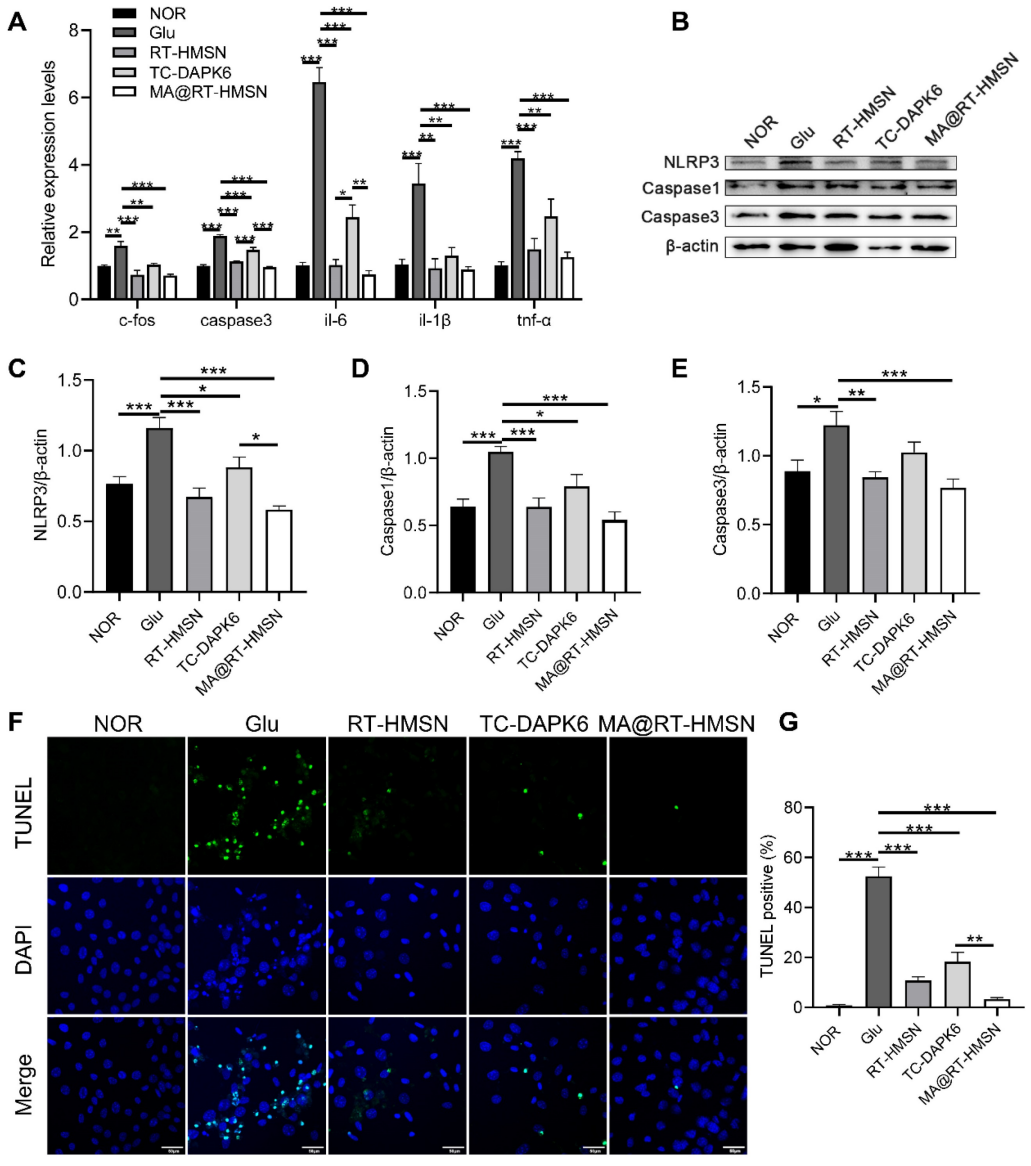
Alleviation of inflammation and apoptosis in glutamate-treated HT22 cells following MA@RT-HMSN incubation. (A) qRT‒PCR analysis of *dapk1*, *c-fos*, *caspase3*,* il-6*, *il-1β* and *tnf-α* expression in the control (NOR), Glu, RT-HMSN, TC-DAPK6 and MA@RT-HMSN groups of HT22 cells (ANOVA; n = 6 in each group). (B) Representative Western blot bands of NLRP3, Caspase1 and Caspase3 in HT22 cells. (C and E) Quantification of the (C) NLRP3, (D) caspase1 and (E) Caspase3 protein levels (ANOVA; n = 5 in each group). (F) Images of TUNEL-stained HT22 cells in the five groups. (G) Quantitative analysis of the percentage of TUNEL-positive cells among the five groups (ANOVA; n = 12 in each group). Scale bar in (F), 50 μm. *P < 0.05, **P < 0.01, ***P < 0.001.

To establish a glutamate-induced excitotoxicity model in HT22 cells *in vitro*, a CCK-8 assay was used to determine cell viability. The results showed that HT22 cell viability decreased gradually with increasing glutamate concentration and was less than 80% after the administration of 20 mM glutamate (Figure S6). We then chose 20 mM as the concentration for glutamate-induced cell injury to investigate the *in vitro* therapeutic effects of RT-HMSN, TC-DAPK6 or MA@RT-HMSN administration. The quantitative real-time polymerase chain reaction (qRT‒PCR) results revealed that 0.1 μM TC-DAPK6 was the optimal concentration for significantly inhibiting the expression of *dapk1* in HT22 cells treated with 20 mM glutamate (Figure S7). Moreover, treatment with RT-HMSNs and MA@RT-HMSNs inhibited the expression of *dapk1* more effectively than treatment with TC-DAPK6 alone (Figure 3A; ANOVA, *P < 0.05, ***P < 0.001). The expression of *c-fos*, a marker of neuronal activity, the apoptosis marker *caspase3* and the inflammatory factors* interleukin-6 (il-6), interleukin-1β (il-1β)* and* tumour necrosis factor-α (tnf-α)* was significantly upregulated at the transcript level following 20 mM glutamate induction. However, after treatment with RT-HMSNs, TC-DAPK6 or MA@RT-HMSNs, the expression levels of these genes decreased significantly (Figure 3A; ANOVA, *P < 0.05, **P < 0.01, ***P < 0.001). Furthermore, glutamate induction significantly upregulated NLRP3, Caspase1 and Caspase3 expression at the protein level in HT22 cells, whereas RT-HMSN, TC-DAPK6 or MA@RT-HMSN treatment significantly decreased their expression (Figure 3B-E; ANOVA, *P < 0.05, **P < 0.01, ***P < 0.001). TUNEL staining revealed that, compared with the control, glutamate caused widespread cell death. However, RT-HMSN, TC-DAPK6 or MA@RT-HMSN treatment significantly attenuated cell death (Figure 3F and G; ANOVA, ***P < 0.001). The above data indicate that RT-HMSN, TC-DAPK6 or MA@RT-HMSN treatment effectively inhibits the expression of *dapk1*, decreases neural activity, alleviates inflammatory responses and reduces apoptosis during cell injury.

### BBB penetration and inflammation-targeting capability of MA@RT-HMSNs

**Figure 4:**
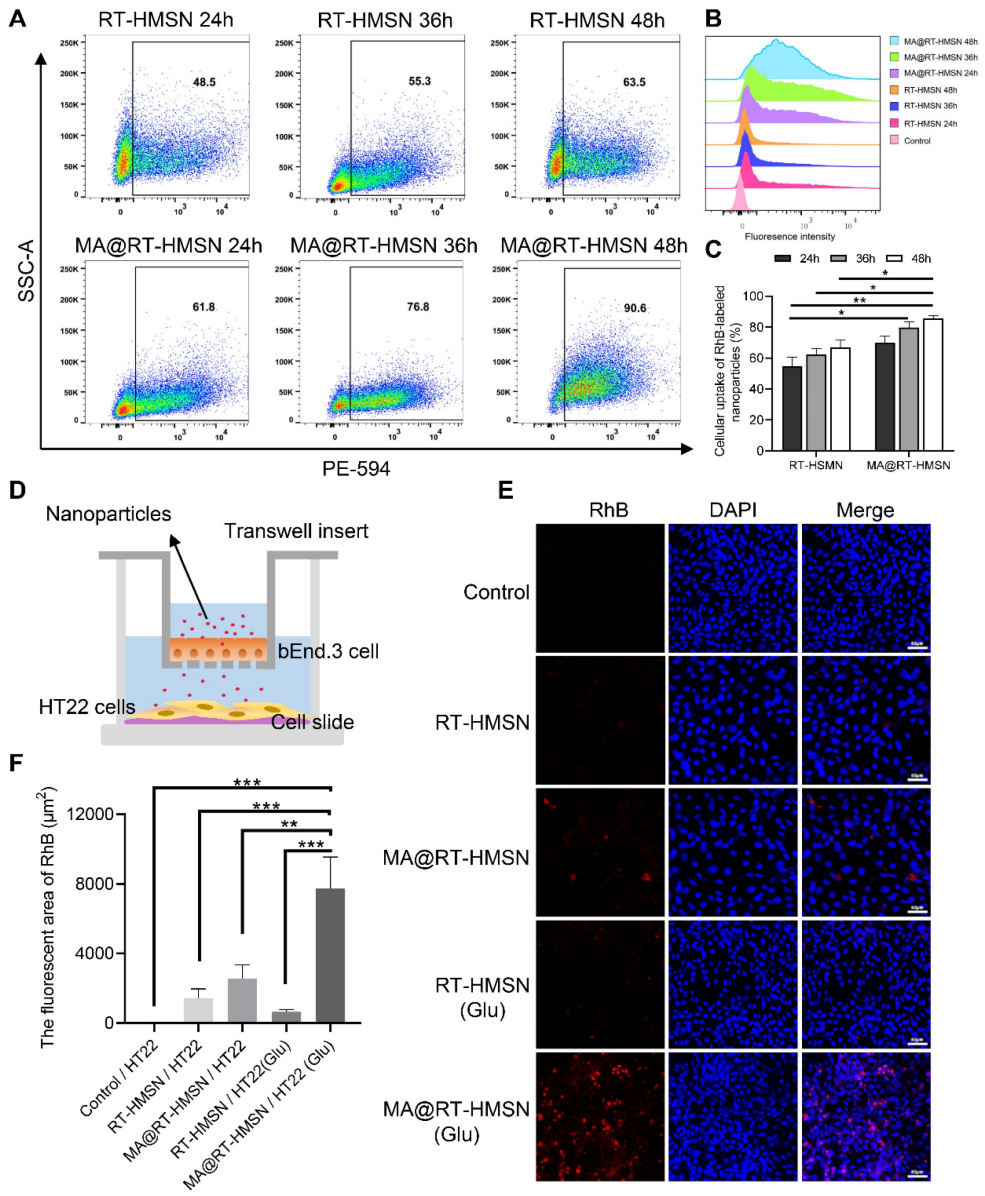
Cellular uptake and *in vitro* BBB penetration of MA@RT-HMSNs. (A-B) Flow cytometry analysis of the cellular uptake of RT-HMSNs and MA@RT-HMSNs after incubation for 24, 36 and 48 h. (C) Quantitative analysis of the flow cytometry of cellular uptake (ANOVA). (D) Schematic diagram of the *in vitro* BBB model. (E) Fluorescence images of HT22 cells in the lower Transwell chambers from the control, RT-HMSN, MA@RT-HMSN, RT-HMSN (Glu) and MA@RT-HMSN (Glu) groups. (F) Quantitative analysis of the red fluorescence intensity in HT22 cells (ANOVA). Scale bar in (A) and (D), 50 µm. *P < 0.05, **P < 0.01, ***P < 0.001.

The *in vitro* fluorescence imaging capability of the nanoparticles was determined by incubating HT22 cells with 25 μg/mL RT-HMSNs or MA@RT-HMSNs. Bright red fluorescence signals were observed in the RT-HMSN and MA@RT-HMSN groups compared with the control group, with no significant attenuation after 48 h. Compared with that in the RT-HMSN group, the red fluorescence intensity in the MA@RT-HMSN group was slightly weaker, which may have been caused by the surface macrophage membrane coating (Figure S8A and B; ANOVA, *P < 0.05, **P < 0.01, ***P < 0.001). The cellular uptake analysis by flow cytometry at different time points (24, 36 and 48 h) revealed a gradual increase in the uptake of RT-HMSNs and MA@RT-HMSNs by HT22 cells (Figure 4A-C). In particular, the cellular uptake of MA@RT-HMSNs was consistently greater than that of RT-HMSNs (Figure 4C; ANOVA, *P < 0.05, **P < 0.01), revealing their strong cell-penetrating ability. Figure 4D shows a schematic diagram of the *in vitro* BBB model. As shown in Figure 4E and F, similar to that in the control group, almost no fluorescence signal was detected in the RT-HMSN and RT-HMSN (Glu) groups, indicating that RT-HMSNs barely crossed the *in vitro* BBB. A few bright red fluorescence signals were found in the cytoplasm of HT22 cells in the MA@RT-HMSN group. However, following glutamate induction, the fluorescence signals in the MA@RT-HMSN (Glu) group were significantly greater than those in the MA@RT-HMSN group (ANOVA, **P < 0.01, ***P < 0.001), suggesting that MA@RT-HMSNs could cross the BBB, especially in the presence of inflammation. Similarly, quantitative analysis by flow cytometry revealed the greatest uptake of MA@RT-HMSNs in HT22 cells stimulated with Glu in the lower chamber (Figure S8C). However, bEnd.3 cells in the upper chamber showed less uptake (Figure S8C), which may be due to increased permeability. Moreover, *ex vivo* imaging revealed that MA@RT-HMSNs accumulated more fluorescence signals in the brains of epileptic mice than in those of normal mice (Figure S9A). Additionally, large amounts of RT-HMSNs and MA@RT-HMSNs accumulated in the liver, kidney and lung, which was due to metabolism and circulation (Figure S9A). The intracranial distribution of MA@RT-HMSNs with red fluorescence signals by microscopy indicated that MA@RT-HMSNs had greater accumulation in both the cortex and hippocampus of brain sections from epileptic mice than in those from normal mice (Figure S9B). In the epileptic mice, the accumulation of MA@RT-HMSNs in the brain gradually increased at 1, 12, and 24 h after injection compared with that in the RT-HMSN group (Figure S9C), suggesting that MA@RT-HMSNs had greater BBB penetration, brain targeting and prolonged intracerebral distribution and stability. The above results not only indicate that MA@RT-HMSNs have excellent imaging capability but also indicate that MA@RT-HMSNs can cross the BBB and target the inflammatory microenvironment.

### Neuronal damage and inflammatory responses were alleviated in kainic acid (KA)-induced acute seizure model mice following MA@RT-HMSN treatment

To investigate the *in vivo* pharmacokinetics of TC-DAPK6 in mice, we first examined the TC-DAPK6 concentrations in the plasma and brain at doses of 2 and 10 mg/kg. The plasma TC-DAPK6 concentration gradually decreased to zero after 8 h (Figure S10A). Moreover, at 2 mg/kg, TC-DAPK6 was almost undetectable in the brain. At 10 mg/kg, the concentration of TC-DAPK6 peaked at 30 min, after which it was rapidly cleared and undetectable after 8 h in the brain (Figure S10B). This result confirms that TC-DAPK6 has a short half-life and a rapid metabolic rate *in vivo*.

**Figure 5:**
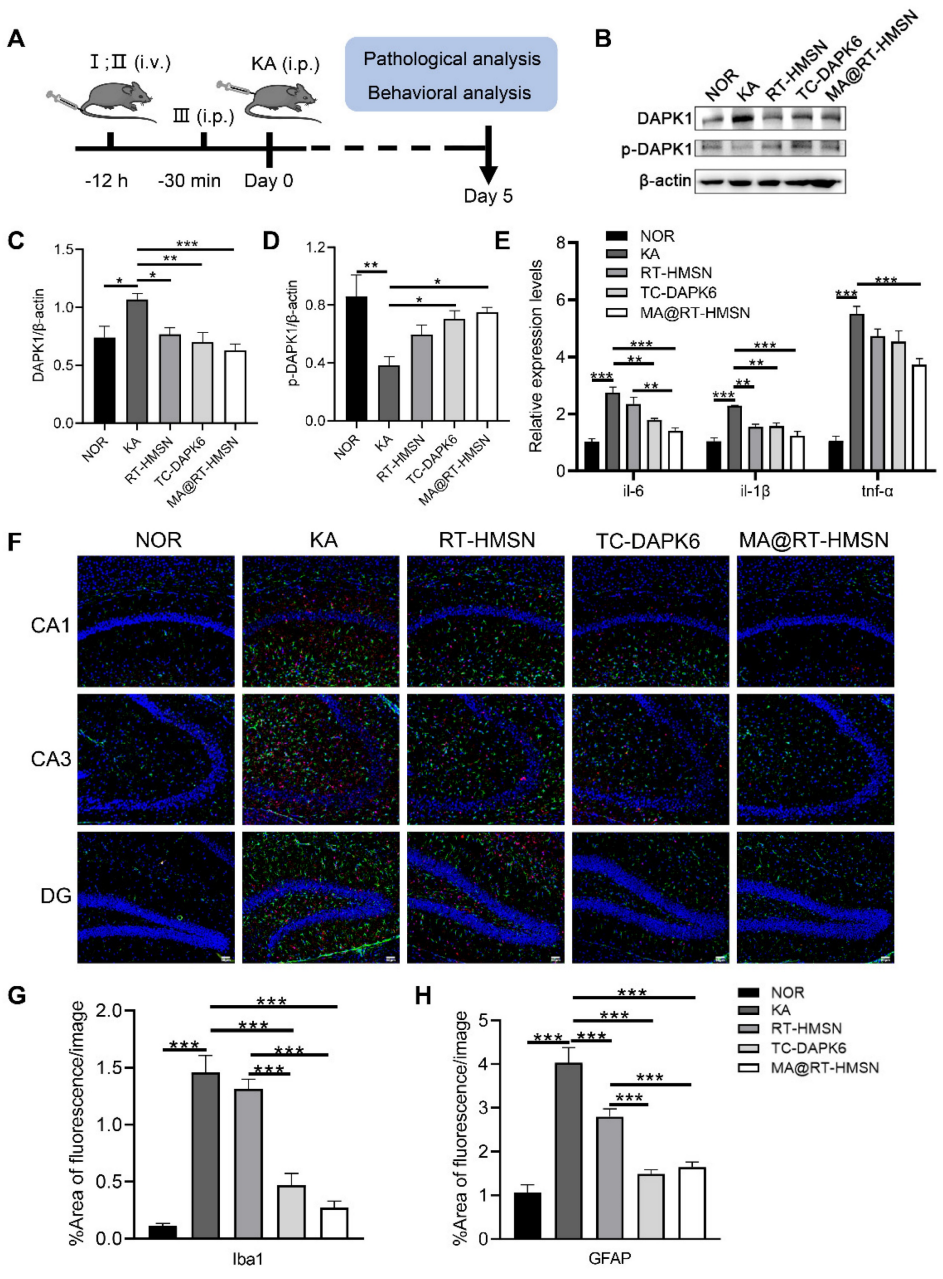
Neuroprotective effects of MA@RT-HMSNs on KA-induced acute seizures. (A) Experimental timeline of three different treatments (I: RT-HMSNs, II: MA@RT-HMSNs and III: TC-DAPK6) and KA injection. (B) Representative Western blot bands showing the protein expression of DAPK1 and p-DAPK1. (C and D) Quantitative analysis of the protein levels of (C) DAPK1 and (D) p-DAPK1. (E) qRT‒PCR analysis of the mRNA expression of *il-6*, *il-1β* and *tnf-α* in control (NOR group), KA-induced (KA group), RT-HMSN-preinjected (RT-HMSN group), TC-DAPK6-preinjected (TC-DAPK6 group) and MA@RT-HMSN-preinjected (MA@RT-HMSN group) mice (ANOVA; n = 6 in each group). (F) Iba1 (red) and GFAP (green) immunostaining of the hippocampus in the three groups. (G and H) Statistical analysis of (G) Iba1- and (H) GFAP-positive cells (ANOVA). Scale bar in (F), 50 µm. *P < 0.05, **P < 0.01, ***P < 0.001.

To assess the *in vivo* therapeutic effects of MA@RT-HMSNs, a mouse model of acute seizures was established via the intraperitoneal administration of KA. The experimental timeline is summarized in Figure 5A. The expression of DAPK1 was significantly increased in the hippocampus of KA-induced mice (Figure 5B and C; ANOVA, *P < 0.05). However, the phosphorylation of DAPK1 at Ser308 was decreased in the KA group, indicating that KA administration increased both DAPK1 expression and activity in the hippocampus (Figure 5B and D; ANOVA, **P < 0.01). Compared with the KA group, MA@RT-HMSN and TC-DAPK6 treatment significantly decreased the expression of DAPK1 and increased the level of DAPK1 phosphorylation at Ser308, which negatively regulated DAPK1 activity in the hippocampus (Figure 5B-D; ANOVA, *P < 0.05, **P < 0.01, ***P < 0.001). Moreover, the expression of the inflammatory cytokines *il-6*, *il-1β* and *tnf-α* was significantly decreased in the MA@RT-HMSN group (Figure 5E; ANOVA, *P < 0.05, **P < 0.01, ***P < 0.001). In this study, Iba1 and GFAP antibodies were used to label microglia and astrocytes, respectively. In the KA group, the Iba1- and GFAP-positive areas increased markedly in the hippocampus (including the CA1, CA3 and dentate gyrus regions); however, they decreased significantly in the MA@RT-HMSN and TC-DAPK6 groups (Figure 5F-H; ANOVA, ***P < 0.001). These findings indicate that MA@RT-HMSN treatment reverses astrogliosis and microgliosis. Additionally, the levels of Ca^2+^ in hippocampal neurons increased after KA induction but decreased after MA@RT-HMSN and TC-DAPK6 treatment (Figure S11; ANOVA, *P < 0.05, **P < 0.01). Moreover, KA promoted the dispersion and loss of granulosa cells in the hippocampal CA3 region, whereas MA@RT-HMSN treatment protected neurons from injury (Figure S12). Furthermore, the KA-induced apoptosis of hippocampal neurons was alleviated after MA@RT-HMSN administration (Figure S13). Collectively, these results revealed the remarkable efficacy of MA@RT-HMSNs in ameliorating neuroinflammation and gliosis and protecting neurons from apoptosis in acute epilepsy compared with RT-HMSNs and TC-DAPK6.

### MA@RT-HMSN treatment improves behaviour and cognition in acute epilepsy model mice

**Figure 6:**
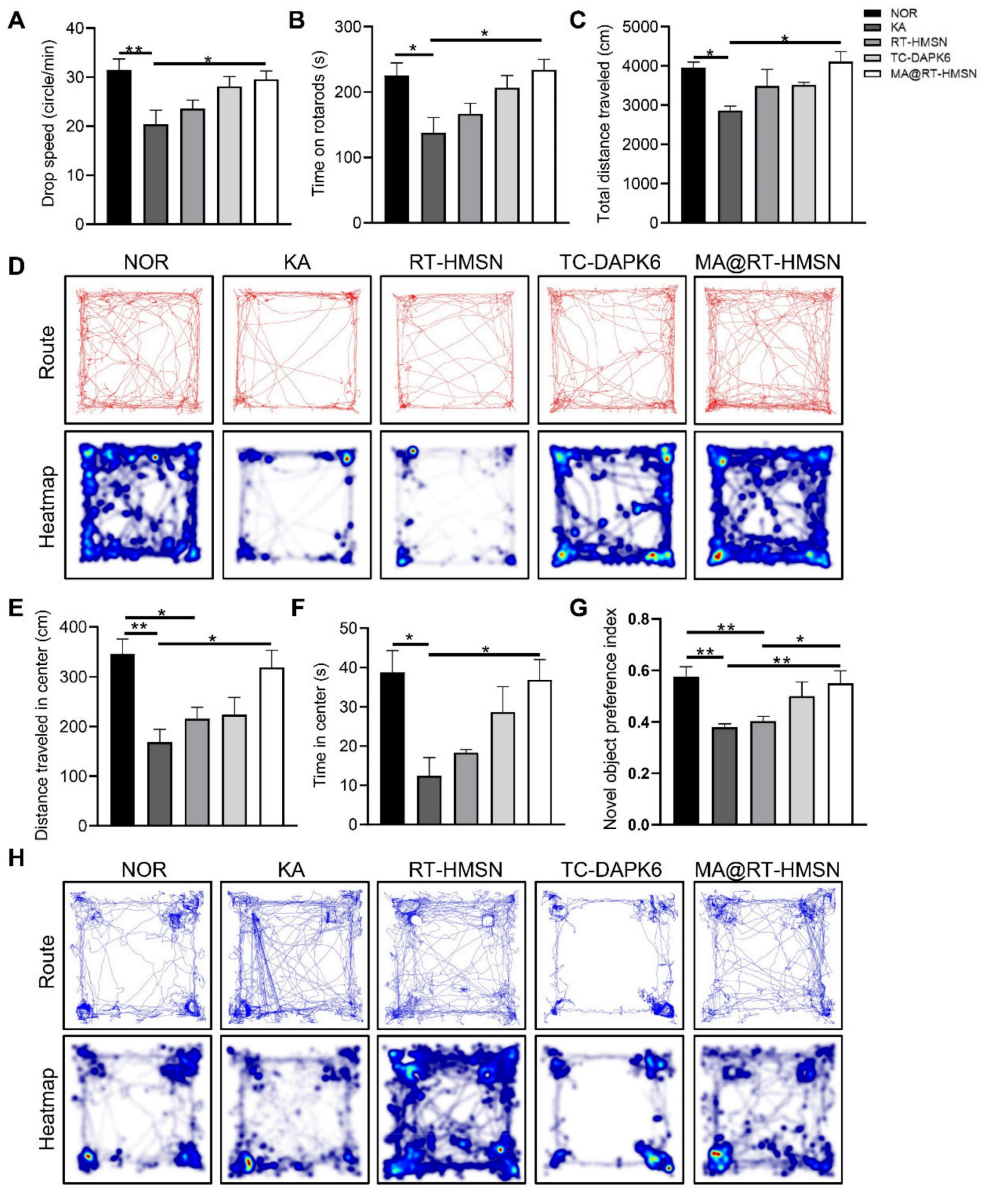
Behavioural analysis of KA-induced acute seizure model mice following MA@RT-HMSN treatment. (A and B) Statistical analysis of (A) time and (B) drop speed in the rotarod test in the NOR, KA, RT-HMSN, TC-DAPK6 and MA@RT-HMSN groups (ANOVA; n = 6 in each group). (C) Statistical analysis of total distance travelled in the open-field test (ANOVA; n = 4 in each group). (D) Typical motion route (top) and heatmap (bottom) showing the results of the open-field test. (E and F) Statistical analysis of (E) distance travelled and (F) time spent in the centre zone by the mice in different groups in the open-field test (ANOVA; n = 4 in each group). (G) Statistical analysis of the preference index for novel objects (ANOVA; n = 4 in each group). (H) Representative trajectory route (top) and heatmap (bottom) showing the results of the novel object recognition test. *P < 0.05, **P < 0.01, ***P < 0.001. NOR, normal control.

To evaluate the functional changes of MA@RT-HMSN treatment, rotarod, open-field and novel object recognition tests were carried out to assess neuromuscular coordination, locomotor activity, and learning and memory ability, respectively. In the rotarod test, the drop speed and the time and distance spent on the rod in the KA group decreased significantly, mildly elevated in the RT-HMSN and TC-DAPK6 groups, and markedly increased in the MA@RT-HMSN group (Figure 6A and B, Figure S14; ANOVA, *P < 0.05, **P < 0.01). Similarly, in the open field test, mice in the KA group showed a significant reduction in the total distance travelled, distance travelled in the centre and time spent in the centre compared with those in the normal control group. However, the exploratory and general activities of the mice in the MA@RT-HMSN group were significantly greater than those in the KA group (Figure 6C-F; ANOVA, *P < 0.05, **P < 0.01). Compared with that of control mice, the preference index for novel objects decreased after KA induction but increased significantly after MA@RT-HMSN administration (Figure 6G and H; ANOVA, *P < 0.05, **P < 0.01). Overall, MA@RT-HMSN treatment obviously improved behavioural activity and cognition and alleviated the anxious depressive state in mice with acute seizures.

### MA@RT-HMSN treatment ameliorates seizure symptoms and neuron impairment in chronic epilepsy model mice

**Figure 7:**
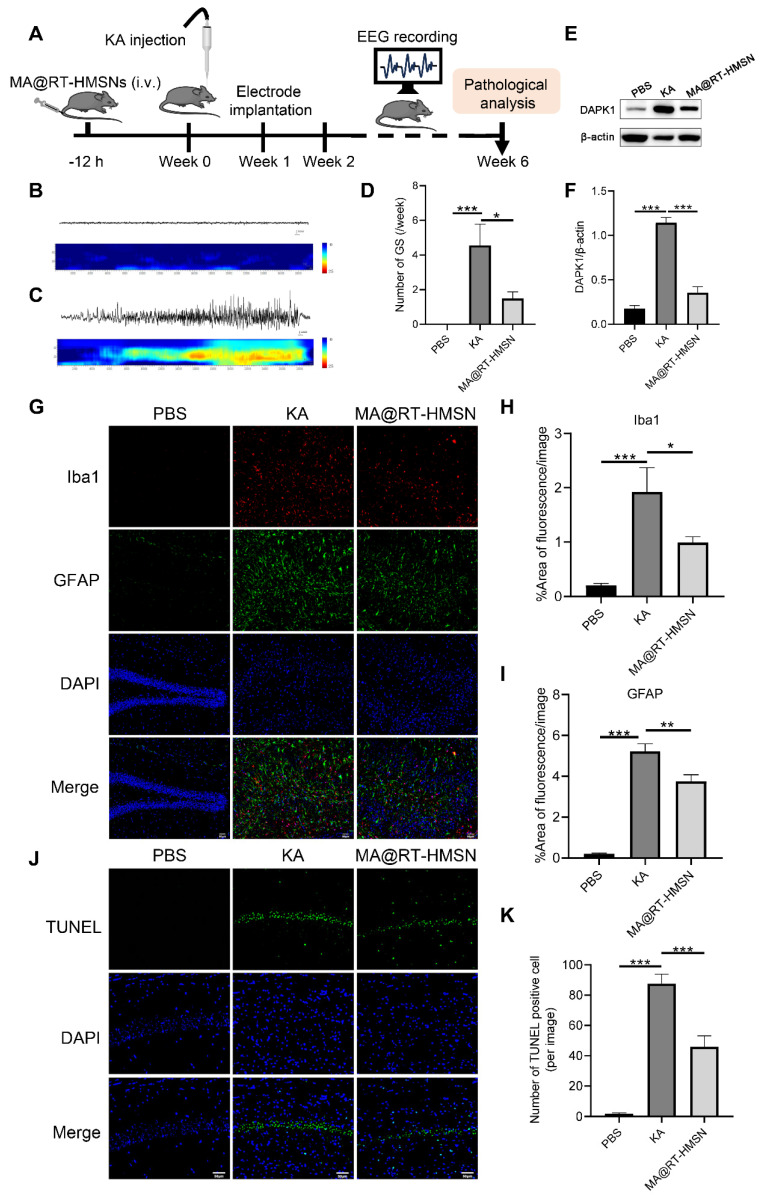
Antiepileptic efficacy of MA@RT-HMSNs in treating KA-induced chronic epilepsy. (A) Schematic illustration of MA@RT-HMSN treatment in KA-induced chronic epilepsy. (B and C) Representative EEGs and corresponding power spectral analysis of the hippocampi of epileptic mice after (B) PBS and (C) KA induction. (D) Statistics of the number of GSs/week in the control (PBS), KA and MA@RT-HMSN groups (n = 3 in each group). (E) Representative Western blot bands showing the protein expression of DAPK1 in the PBS, KA and MA@RT-HMSN groups. (F) Quantitative analysis of the protein level of DAPK1 (ANOVA; n = 5 in each group). (G-I) Images of (G) immunostaining and statistical analysis of (H) Iba1- and (I) GFAP-positive cells in the hippocampus in the three groups (ANOVA; n = 3 in each group). (J-K) Images of (J) TUNEL staining and statistical analysis of (K) the numbers of TUNEL-positive cells in the hippocampus in the three groups (ANOVA; n = 3 in each group). Scale bar in (G) and (J), 50 µm. *P < 0.05, **P < 0.01, ***P < 0.001.

The experimental schedule for the chronic epilepsy model is displayed in Figure 7A. Electroencephalogram (EEG) recordings were taken from 2 to 6 weeks. The representative EEG traces and corresponding power spectra of the baseline and generalized tonic-clonic seizures (GSs) are shown in Figure 7B and C. Following MA@RT-HMSN treatment, spontaneous seizures were inhibited, and the number of GSs significantly decreased (Figure 7D; ANOVA, *P < 0.05, **P < 0.01, ***P < 0.001). DAPK1 protein upregulation induced by KA was also reduced (Figure 7E and F; ANOVA, ***p < 0.001); in addition, the areas of Iba1-positive and GFAP-positive signals were markedly decreased in the hippocampus (Figure 7G-I; ANOVA, *P < 0.05, **P < 0.01, ***P < 0.001). TUNEL staining revealed that MA@RT-HMSN treatment relieved cell apoptosis in the hippocampus caused by KA injection (Figure 7J and K; ANOVA, ***P < 0.001). Taken together, the specific inhibition of DAPK1 by MA@RT-HMSNs ameliorates spontaneous seizures, prevents astrogliosis and microgliosis and reduces apoptosis, thus exerting neuroprotective and therapeutic effects on chronic epilepsy.

## Discussion

Accumulating evidence indicates that there is a positive feedback loop between seizures and inflammatory responses 30. During epileptogenesis, cytokine receptors and proinflammatory factors are upregulated in neurons, microglia, astrocytes and endothelial cells, triggering a subsequent wave of inflammation 31. Conversely, CNS inflammation enhances neuronal excitability by modulating ion channel and glutamate release in neurons and glial cells or by altering the structure of neural networks, leading to an increasing tendency to develop seizures 32, 33. In this study, we established a glutamate-induced cell injury model that simulates hippocampal neuron damage in epilepsy 34. We found that glutamate stimulation significantly increased the expression of inflammatory cytokines, apoptosis-related factors and *dapk1* in HT22 cells. TC-DAPK6 was selected for the pharmacological inhibition of DAPK1.* In vitro* experiments demonstrated that TC-DAPK6 treatment not only effectively inhibited the expression of *dapk1* but also decreased neural activity, alleviated inflammatory responses and reduced apoptosis. Therefore, TC-DAPK6 has emerged as a promising antiepileptic drug designed to combat neuroinflammation and neuronal damage associated with epilepsy rather than merely symptomatic control of seizures, as with traditional treatments 35.

However, the *in vivo* application of TC-DAPK6 is limited because of three concerns. First, a pharmacokinetic test confirmed that TC-DAPK6 has a short half-life and a rapid metabolic rate *in vivo*. Second, TC-DAPK6 is a hydrophobic drug that is unable to maintain its effective concentration *in vivo*. Finally, DAPK1 is widely expressed in different tissues, including the heart, lung, spleen and brain, and is involved in a variety of physiological and pathological events 8.

In this study, we developed a novel kind of macrophage membrane-biomimetic nanoparticle, namely, MA@RT-HMSNs. Two molecules, TC-DAPK6 and RhB, were encapsulated simultaneously in the pores of HMSNs by physical adsorption, resulting in the preparation of nanoscale RT-HMSNs 23, 24. The nanoscale channels efficiently dispersed hydrophobic TC-DAPK6 molecules, which changed the crystalline form into the amorphous state, thus improving the intracellular solubility and stability of TC-DAPK6 36. Furthermore, RT-HMSNs automatically enabled the slow and steady release of TC-DAPK6 from the porous structure of the HMSNs 21. Therefore, RT-HMSNs exhibit greater inhibitory and therapeutic effects than free TC-DAPK6 does *in vitro*.

BBB permeability is a prerequisite for the effective delivery of nanoparticles to the brain 37. Despite the small size of nanoparticles, this does not necessarily mean that they can cross the BBB. To improve the efficiency of BBB penetration, current strategies mainly include nanoparticle surface modification and cell membrane coating 27, 38. Surface modification involves coating or combining polymer molecules to increase their hydrophilicity and biocompatibility, which in turn improves their BBB permeability 39. Additionally, nanoparticles are functionalized with specific ligands, such as angiopep-2, which relies on a single receptor-mediated mechanism to specifically bind low-density lipoprotein receptor-related protein-1 on the BBB to drive transendocytosis and promote drug entry into the brain 40, 41. The cell membrane coating endows nanoparticles with natural multitargeting capabilities. Particularly, macrophages express specific membrane receptors, such as lymphocyte function-associated antigen-1, alpha M integrin-1, toll-like receptors and chemokine receptors, which facilitate nanoparticles crossing BBB and promote their chemotaxis and accumulation toward inflammatory sites 42, 43.

The epileptogenesis process is usually accompanied by inflammation, which contributes to the breakdown and opening of the BBB, allowing the recruitment of circulating macrophages to the brain 44-46. In this study, RT-HMSNs were functionalized with coated macrophage membranes to prepare biomimetic MA@RT-HMSNs. The *in vitro* BBB model established by a coculture system of bEnd.3 cells and glutamate-treated HT22 cells demonstrated that MA@RT-HMSNs successfully crossed the BBB under conditions of inflammation induced by cell injury 47. Moreover, to assess the *in vivo* BBB permeability and inflammation-targeting capability of MA@RT-HMSNs, a KA-induced mouse seizure model was constructed to simulate hippocampal injury in individuals with epilepsy 48, 49. Our study demonstrated that KA induced the upregulation of inflammatory cytokine expression and gliosis in the mouse hippocampus. The *ex vivo* imaging results demonstrated that MA@RT-HMSNs could cross the BBB even under physiological conditions, but many more fluorescence signals accumulated in the brains of mice with acute epilepsy. Moreover, the macrophage membrane prevents the loaded drug from leaking and prematurely releasing until it is internalized into cells and transferred to acidic lysosomes 21, 50. Compared with RT-HMSNs, macrophage membrane encapsulation significantly promoted the cellular uptake of MA@RT-HMSNs. Additionally, the accumulation of MA@RT-HMSNs in the epileptic brain gradually increased after injection. In acute and chronic epilepsy, MA@RT-HMSNs had a greater therapeutic effect than RT-HMSNs or TC-DAPK6. The coated macrophage membrane endowed the MA@RT-HMSNs with unique macrophage-like properties, which not only improved BBB permeability and inflammation-guided targeting but also improved the bioavailability and stability of TC-DAPK6. More importantly, the macrophage membrane coating enhances the biocompatibility of nanoparticles *in vitro* and* in vivo*.

The consequences of targeted DAPK1 inhibition were determined based on three aspects. First, MA@RT-HMSN administration significantly inhibited DAPK1 expression, promoted autophosphorylation at Ser308 to negatively regulate DAPK1 activity and reduce Ca^2+^ levels in acute epilepsy. It has been shown that DAPK1 phosphorylation plays an important role in neural cell death, especially dephosphorylation at Ser308, which can positively regulate the activation of DAPK1 9. Activated DAPK1 interacts with N-methyl-D-aspartate (NMDA) receptors in neurons to overexcitate NMDA receptors, resulting in excessive Ca^2+^ influx and excitotoxic damage in nerve cells 11. Second, MA@RT-HMSNs significantly inhibited inflammatory responses, gliosis and apoptosis in acute and chronic epilepsy. Reactive gliosis releases various inflammatory factors and reactive oxygen species, which further promote the activation of surrounding resting glial cells, causing persistent oxidative stress and an inflammatory microenvironment 51. Moreover, the release of cytokines by microglia and astrocytes increases the influx of neuronal calcium, leading to neuronal hyperexcitability 52. In turn, inflammatory factors stimulate astrocytes to release glutamate, reduce their uptake of potassium ions and glutamate and increase the extraneuronal glutamate concentration, eventually triggering neuronal hyperexcitability and nerve damage 33, 53. Finally, MA@RT-HMSN administration alleviated seizures and restored locomotion, anxiety, depression and cognitive impairment. Hippocampal neurons are highly susceptible to epileptic discharges and excitotoxic damage, and long-term seizures or SE can lead to the death of hippocampal neurons, causing cognitive impairment in epilepsy 54. In this study, targeting DAPK1 alleviated seizures primarily by reducing inflammation and nerve damage. We believe that future studies on TC-DAPK6 and antiepileptic drug-coloaded biomimetic nanoparticles will not only overcome the poor BBB permeability, poor solubility and short half-life of AEDs but also exert synergistic therapeutic effects on epilepsy.

## Conclusion

In summary, the prepared MA@RT-HMSNs exhibited BBB permeability and inflammation-guided targeting and enabled the precise delivery of TC-DAPK6 to epileptic foci. Targeted inhibition of DAPK1 decreases neural activity, ameliorates inflammatory responses, prevents gliosis, reduces apoptosis, and promotes the recovery of cognition, thus exerting neuroprotective and therapeutic effects on epilepsy. Therefore, MA@RT-HMSNs, a novel kind of macrophage membrane-biomimetic nanoparticle, offer a promising approach for epilepsy treatment.

## Materials and methods

### Synthesis of RT-HMSNs and MA@RT-HMSNs

For the synthesis of the RT-HMSNs, 2 mg of HMSNs (XFNANO Biological Technology Co., Ltd., Nanjing, China), 2 mg of TC-DAPK6 (MedChemExpress LLC., Shanghai, China) and 1 mg of RhB (Macklin, Shanghai, China) were dispersed in 5 mL of anhydrous ethanol by ultrasonic processing and then stirred at room temperature for 12 h. The obtained mixture was centrifuged, and the supernatant was collected to determine the encapsulation efficiency of TC-DAPK6 and RhB in HMSNs by HPLC (LC20ADXR, Shimatsu, Japan) and UV‒vis spectroscopy (Shimadzu, Japan), respectively. The product was washed three times with ethanol and water and then dried for 10 h to obtain solid RT-HMSNs samples. In addition, under the same conditions, T-HMSNs were synthesized at a 1:1 mass ratio of HMSNs to TC-DAPK6, and R-HMSNs were synthesized at a 2:1 mass ratio of HMSNs to RhB.

Macrophage membranes were extracted from the RAW264.7 mouse macrophage line purchased from Procell Life Science & Technology Co., Ltd. (Wuhan, China) using a cell membrane protein extraction kit (Beyotime, Shanghai, China) according to the manufacturer's protocol. The MAs were prepared by repeatedly extruding through 400- and 200-nm polycarbonate porous membranes using an Avanti mini extruder (Avanti Polar Lipids, AL, USA). RT-HMSNs were mixed with MAs at a weight ratio of 2:1, followed by extrusion through a 200-nm polycarbonate membrane at least 10 times. The excess cell membrane vesicles were removed by centrifugation, and the final macrophage membrane-coated RT-HMSNs (MA@RT-HMSNs) were obtained.

### Characterization

The particle size and morphology of the nanoparticles were characterized by TEM (Hitachi, Japan). The hydrated particle size and zeta potential were determined via dynamic light scattering (DLS; Zetasizer Nano ZS90). TGA was carried out on a TG-DTA8122 thermal analyser (Rigaku, Japan) from 30 to 800 °C (10 °C/min) in a controlled atmosphere to determine the composition and thermal stability. PXRD measurements were obtained by SmartLab 9KW (Rigaku, Japan) scanning at a range of 5-60 °C (5 °C/min) to analyse the crystallographic structure of the prepared samples. Proteins from the macrophage lysates, macrophage membrane fragments, and MA@RT-HMSNs were extracted, separated by SDS‒PAGE and characterized by Coomassie blue staining (Solarbio, Beijing, China). Western blot experiments were performed to verify the presence of the cell membrane proteins CD11b and F4/80 in MA@RT-HMSNs.

### High-performance liquid chromatography (HPLC)

To quantify TC-DAPK6 in HMSNs, HPLC was used to determine the TC-DAPK6 encapsulation efficiency (EE) using a YMC-Triart C18 column (100×2.1 mm; 3 µm). Acetonitrile/water (30/70, v/v) containing 0.1% methane acid was used as the mobile phase at a flow rate of 0.6 mL/min. The equation for EE was as follows:



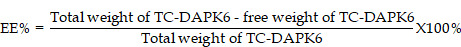



### Cytotoxicity

The mouse hippocampal cell line HT22 and the mouse brain microvascular endothelial cell line bEnd.3 were obtained from ATCC (Mansas, VA, USA) and seeded into 96-well plates at a density of 8×10^3^ cells/well for 24 h. The cells were then incubated in 100 μL of medium containing different concentrations (0, 5, 12.5, 25, 50 or 100 μg/mL) of RT-HMSNs or MA@RT-HMSNs for 24 h. A CCK-8 kit (MedChemExpress) was used to evaluate cytotoxicity. The absorbance at 450 nm was measured with a microplate reader (NanoQuant, Tecan, Switzerland), and the cell viability was calculated. Each sample was assayed in at least three parallel replicates.

### *In vitro* biological imaging

To detect the bioimaging ability of the nanoparticles, HT22 cells were seeded in 24-well plates with cell slides for 24 h. The cells were then incubated with medium containing 25 μg/mL of RT-HMSNs or MA@RT-HMSNs for 24, 36 or 48 h. The cell slides were collected and mounted with ProLong anti-fade mounting media supplemented with DAPI (Abcam, Cambridge, MA, USA). Red fluorescence of RhB and blue fluorescence of the DAPI stain were observed under a Nikon ECLPSE TI confocal microscope (Japan) with 559 nm and 405 nm excitation, respectively.

### Cellular uptake of nanoparticles

HT22 cells were seeded in 6-well plates at a density of 5×10^5^ cells/well for 24 h. The cells were then incubated with RT-HMSNs or MA@RT-HMSNs at a concentration of 25 μg/mL for 24, 36 or 48 h. The cells were washed three times with PBS, digested and collected. Flow cytometry (LSRFortessa, BD Biosciences, San Jose, CA, USA) was used to analyse the fluorescence of the RhB-labelled nanoparticles.

### Cell injury and treatment

HT22 cells were seeded into 96-well plates at a density of 8×10^3^ cells/well and stimulated with different concentrations (0, 5, 10, 15, 20 and 25 mM) of L-glutamic acid (glutamate; MedChemExpress) for 24 h. Cell viability was assessed by the CCK-8 assay. HT22 cells were seeded into 6-well plates at a density of 8×10^5^ cells/well and treated with TC-DAPK6 at concentrations of 0, 0.1, 1 and 10 μM and glutamate at 20 mM for 24 h. qRT‒PCR was then used to measure the expression of *dapk1* in HT22 cells.

To study the protective effects of TC-DAPK6 on glutamate-induced cell injury, HT22 cells were seeded into 6-well plates and divided into five groups. In the normal control (NOR) group, the HT22 cells were cultured in complete culture medium; in the Glu group, the HT22 cells were cultured in medium supplemented with 20 mM glutamate; in the RT-HMSN group, the HT22 cells were pretreated with 25 μg/mL RT-HMSNs for 12 h, followed by 20 mM glutamate for 24 h; in the MA@RT-HMSN group, the HT22 cells were pretreated with 25 μg/mL MA@RT-HMSNs for 12 h, followed by 20 mM glutamate for 24 h; and in the TC-DAPK6 group, the HT22 cells were cultured in medium supplemented with 0.1 μM TC-DAPK6 and 20 mM glutamate for 24 h.

### *In vitro* BBB model

A bEnd.3 and HT22 cell coculture model was established to evaluate the *in vitro* BBB penetration and targeting ability of MA@RT-HMSNs. Briefly, bEnd.3 cells were seeded into the upper chamber of a Transwell insert (pore size 0.4 μm, diameter 6.5 mm) in a 24-well plate at a density of 3×10^4^ cells/well. The culture medium was changed every other day, and the transendothelial electrical resistance (TEER) was measured every day using an EVOM2 instrument and an STX2 electrode (World Precision Instruments Inc., Sarasota, FL, USA). When the TEER reached 200 ohm·cm^-2^, the bEnd.3 monolayer in the upper chamber was transferred to another Transwell in which HT22 cells were seeded on cell slides in the bottom chamber. The medium in the upper chamber was replaced with medium containing RT-HMSNs or MA@RT-HMSNs. HT22 cells were treated with medium or 20 mM glutamate in the bottom chamber. The bEnd.3 and HT22 cells were then cocultured for 24 h. The cell slides were collected, and the red fluorescent signals of the HT22 cells were observed under a Nikon ECLPSE TI confocal microscope. In addition, bEnd.3 and HT22 cells were collected to analyse the cellular uptake of RT-HMSNs or MA@RT-HMSNs using flow cytometry.

### Animals

Specific pathogen-free male C57BL/6J mice (6 to 8 weeks) were purchased from Beijing Charles River Laboratory Animal Technology Co., Ltd. (Beijing, China). The mice had access to sterile food and water *ad libitum* and were housed in cages at 22-25 °C with a relative humidity of 40-70% under a 12 h alternating light and dark cycle. All animal experiments were performed in compliance with the Laboratory Animal Welfare and Ethics Committee of Xuanwu Hospital, Capital Medical University (permission number: XW20220906-1) and the National Institutes of Health Guide for the Care and Use of Laboratory Animals.

### Determination of TC-DAPK6 concentrations in the blood and brain tissues of mice

Mice were intraperitoneally injected with TC-DAPK6 at doses of 2 mg/kg or 10 mg/kg. The mice were deeply anaesthetized by intraperitoneal injection of 50 mg/kg sodium pentobarbital. Blood was collected from the retro-orbital sinus. The plasma samples were collected and diluted 1:1 with an internal standard (IS). Then, 50 μL of acetonitrile was added, and the mixture was vortexed, followed by centrifugation at 14,000 rpm for 10 min at 4 °C. The supernatant was mixed with ultrapure water for liquid chromatography‒tandem mass spectrometry analysis. Brain tissue was harvested after cardiac perfusion with 0.9% saline. Tissue homogenization was performed by adding acetonitrile at a brain weight/volume ratio of 250 g/L. A total of 10 μL of tissue homogenate sample was taken and added to 10 μL of IS solution. The next detection step involved the plasma sample detection procedure as described above.

### KA-induced seizure model and epilepsy treatment

The acute seizure mouse model was induced by intraperitoneal injection of KA (Abcam) at a dose of 30 mg/kg. The seizure severity was recorded according to the modified Racine scoring system 55. Mice exhibiting more than two consecutive grade 4 or higher seizures were considered successfully modelled. In the RT-HMSN and MA@RT-HMSN groups, the mice were preinjected with RT-HMSNs or MA@RT-HMSNs at a dose of 3 mg/kg before KA injection and then injected every two days. In the TC-DAPK6 group, the mice were preinjected with TC-DAPK6 at a dose of 10 mg/kg before KA injection and then administered TC-DAPK6 once a day.

A chronic epilepsy mouse model was established by injecting KA into the dorsal hippocampus. Briefly, the mice were anaesthetized and mounted in a stereotaxic apparatus. The location of the hippocampal CA1 region (antero-posterior, -2.0 mm; lateral, -1.5 mm; ventral, -1.8 mm) was determined from the bregma of the mouse brain, and KA solution (1 ng/nL, 200 nL) was slowly injected using a microsyringe. After each injection, the syringe was left in place for 5 min before withdrawal. The control group was injected with an equal amount of PBS (0.01 M, pH 7.4) at the same position. In the MA@RT-HMSN group, the mice were pretreated with MA@RT-HMSNs at a dose of 3 mg/kg via the tail vein before intrahippocampal injection of KA and then injected with MA@RT-HMSNs every two days until the tissue was collected after 6 weeks.

### EEG recording

In the chronic epilepsy model, one week after KA injection, the mice were anaesthetized and placed on a stereotaxic apparatus to implant electrodes into the hippocampal CA1 region. EEG recordings were collected from freely moving mice, which were synchronized with video recordings continuously for 8 h/day. Seizure events were analysed by combining video seizure behaviour and EEG activity. A GS was identified as a train of spike discharges lasting more than 15 s, with an amplitude of at least 2× the initial baseline amplitude, accompanied by obvious manifestations of a GS.

### Biochemical analysis of blood

The mice were anaesthetized, and blood samples were collected from the retro-orbital sinus for measurement of ALT, AST, CR and BUN levels.

### H&E staining

The brain, heart, liver, spleen, lung and kidney were dissected, fixed in 4% PFA overnight at 4 °C, embedded in paraffin and serially sectioned at a thickness of 4 µm. H&E staining was performed using standard protocols. Images of H&E-stained sections were captured with a BX51 Olympus optical microscope (Tokyo, Japan).

### Immunofluorescence staining

Mouse brains were fixed in 4% PFA overnight at 4 °C and dehydrated with 15% and 30% sucrose solutions for 24 h. The samples were embedded in optimal cutting temperature compound (Sakura Finetek; Torrance, CA, USA) and sectioned at a thickness of 8 µm. The primary antibodies used were rabbit anti-NeuN (1:500; Abcam), rabbit anti-GFAP (1:500; Abcam), and goat anti-Iba1 (1:500; Abcam). The secondary antibodies used included Alexa Fluor 488 (1:500; Thermo Fisher Scientific, Waltham, MA, USA) and Alexa Fluor 647 (1:500; Thermo Fisher Scientific). DAPI was used to label the nuclei. Images of the immunostained sections were captured using a confocal microscope (MICA, Leica, Germany).

### *In vivo* fluorescence imaging

RhB was used as a fluorescent probe to evaluate the BBB penetration of the nanoparticles *in vivo*. Normal mice or KA-induced acute epileptic mice were administered MA@RT-HMSNs via the tail vein. One hour later, the mice were deeply anaesthetized. The brain, liver, lung, kidney, heart and spleen were harvested for *ex vivo* imaging. The fluorescent signals (excitation, 535 nm; emission, 580 nm) were captured using an IVIS Spectrum (Revvity; Waltham, MA, USA). In addition, the brain was collected, sectioned at a thickness of 30 µm, and the nuclei were labelled with DAPI. The intracranial microscopic distribution of MA@RT-HMSNs was observed by a confocal microscope. For the targeted accumulation of RT-HMSNs and MA@RT-HMSNs in the brain of acute epilepsy, KA-induced mice were injected with RT-HMSNs or MA@RT-HMSNs via the tail vein. The brains were harvested at 1, 12 and 24 h for *ex vivo* imaging using an IVIS Spectrum system (Revvity).

### Measurement of Ca^2+^ concentration

The concentration of Ca^2+^ was measured with a calcium colorimetric assay kit (Beyotime). Hippocampal tissue was mixed with lysis buffer and homogenized at 4 °C. The supernatant was obtained by centrifugation and incubated with a chromogenic agent solution at room temperature for 10 min in the dark. A microplate reader was used to measure the absorbance at 575 nm. The concentration of Ca^2+^ was calculated according to the standard curve.

### TUNEL assay

A TUNEL apoptosis assay kit (Beyotime) was used to investigate neuronal death in HT22 cells *in vitro* or in the mouse hippocampus according to the manufacturer's protocol. HT22 cell slides or brain sections were mounted with ProLong anti-fade mounting media with DAPI and photographed using a confocal microscope (MICA, Leica, Germany) at excitation wavelengths of 488 nm and 405 nm, respectively.

### qRT‒PCR

Total RNA was extracted from hippocampal tissue or HT22 cells using a cell/tissue total RNA isolation kit (Vazyme, Nanjiing, China). Total RNA (1 µg) was used for the synthesis of cDNA with 1st Strand cDNA Synthesis SuperMix from Yeasen Biotech (Yeasen, Shanghai, China). qRT‒PCR was carried out using an LC480 system (Roche, Switzerland). The reaction program was set up as follows: predenaturation at 95 °C for 5 min, followed by 40 cycles of denaturation at 95 °C for 10 s, annealing at 60 °C for 20 s and extension at 72 °C for 20 s. The cycle threshold (CT) values were normalized to those of β-actin, and the 2^-ΔΔCT^ method was used for quantitative analysis. The primer sequences are listed in Table S1, Supporting Information.

### Western blot analysis

Total proteins were extracted from hippocampal tissues or HT22 cells using lysis buffer (CWBIO, Beijing, China). The total protein concentration was calculated according to the BCA protein quantitative assay kit (CWBIO). The following primary antibodies were used in this study: rabbit anti-F4/80 antibody (1:1000; Abcam), rabbit anti-CD11b (1:1000; Abcam), rabbit anti-NLRP3 (1:1000; Affinity Biosciences, Cincinnati, OH, USA), rabbit anti-Caspase1 (1:1000; Proteintech, Chicago, IL, USA), rabbit anti-Caspase3 (1:1000; Cell Signaling Technology, Danvers, MA, USA), mouse anti-DAPK1 (1:1000; Proteintech) and rabbit anti-phospho-DAPK1 (p-DAPK1; 1:1000; Affinity Biosciences). Mouse anti-β-actin (1:20000; Proteintech) was used as a loading control. The secondary antibodies used were anti-horseradish peroxidase (HRP)-rabbit (1:5000; Abcam) and HRP-anti-mouse (1:5000; Abcam) antibodies. Optical density analysis was performed using ImageJ software (ver 1.8.0, National Institute of Health, Bethesda, MD, USA), and the data were normalized to the internal standard β-actin.

### Behavioural tests

All behavioural tests were performed in a soundproof, quiet and dim room between 09:30 and 16:30. The mice were allowed to adapt to the environment for one day before the behavioural experiments. The tests were monitored and recorded by a camera and analysed with Noldus Ethovision XT17 software (Noldus Information Technology, Wageningen, Netherlands).

For the rotarod test, the mice were placed on the rotarod (KEW, Nanjing, China) apparatus for 5 min to acclimate for balance. Then, the cylinder was rotated at a blank speed of 4 rpm for 30 s, accelerated from 0 to 40 rpm for 5 min and then rotated at a fixed speed for another 1 min. Each mouse was subjected to three trials at 20 min intervals. The latency time, drop speed and travel distance of the mice remaining on the rod were recorded during each trial.

For the open-field test, a box (50 cm × 50 cm × 50 cm) with an open top and white bottom was used as the test site. The test was set for 10 min. The motion trail of each mouse was captured, and the total distance travelled, central area distance travelled and cumulative total time spent in the centre were recorded.

For the novel object recognition test, the experiment was divided into three stages: an adaptation period, a training period and a testing period. First, the mice were placed in an empty box and allowed to freely move and explore for 10 min. Second, two identical objects were placed in the box, and the mice were placed inside the box to explore for 10 min. Finally, after 1 h, one of the objects was replaced by a novel object of a different shape, and the behaviours of the mice in the box were recorded for 10 min. Mouse preference for novel and familiar objects was calculated based on the preference index for novel objects (time spent exploring novel objects/total time spent exploring novel and familiar objects).

### Statistical analysis

All of the statistical analyses were performed with GraphPad Prism software (version 8.0; GraphPad Software, La Jolla, USA). The data are presented as the mean ± standard error of the mean (SEM). Differences among the experimental groups were analysed by one-way analysis of variance (ANOVA) followed by Tukey's post hoc test. A P value less than 0.05 was considered to indicate statistical significance.

## Supplementary Material

Supplementary figures and table.

## Abbreviations

AEDs: antiepileptic drugs
BBB: blood‒brain barrier
DAPK1: death-associated protein kinase 1
HMSNs: hollow mesoporous silica nanocarriers
CNS: central nervous system
MSNs: mesoporous silica nanoparticles
RhB: rhodamine B
DAPI: 4′,6-diamidino-2-phenylindole
TEM: transmission electron microscopy
PDI: polydispersity index
MA: macrophage membrane
PXRD: powder X-ray diffraction
TGA: thermogravimetric analysis
UV‒vis: UV‒visible
HPLC: high-performance liquid chromatography
PAGE: polyacrylamide gel electrophoresis
ALT: alanine aminotransferase
AST: aspartate aminotransferase
CR: creatinine
BUN: blood urea nitrogen
H&E: haematoxylin and eosin
IL-6: interleukin-6
IL-1β: interleukin-1β
TNF-α: tumour necrosis factor-α
qRT‒PCR: quantitative real-time polymerase chain reaction
KA: kainic acid
EEG: electroencephalogram
GSs: generalized tonic-clonic seizures
NMDA: N-methyl-D-aspartate
DLS: dynamic light scattering
EE: encapsulation efficiency
TEER: transendothelial electrical resistance
IS: internal standard
NOR: normal control
