# Supplementary material for: Macrophage membrane‒biomimetic nanoparticles target inflammatory microenvironment for epilepsy treatment
Source: Theranostics. 2024 Oct 7;14(17):6652–70. doi: 10.7150/thno.99260 (PMC11519803; doi:10.7150/thno.99260)
Supplement: Supplementary file 1 — Supplementary figures and table. [file thnov14p6652s1.pdf]

Supplementary material

Macrophage membrane–biomimetic nanoparticles target inflammatory microenvironment for epilepsy treatment

Chao Geng[1,2], Xinghui Ren[3], Peipei Cao[1,2], Xiaoqi Chu[1,2], Penghu Wei[2], Quanlei Liu[2], Yongchang Lu[2], Bin Fu[2], Wenyou Li[3,*], Yuhao Li[4,5,*], Guoguang Zhao[2,6,7,*]

1 Optometry Institute, School of Medicine Nankai University, Tianjin, 300071, China

2 Department of Neurosurgery, Xuanwu Hospital Capital Medical University, Beijing Municipal Geriatric Medical Research Center, Beijing, 100053, China

3 College of Chemistry, Research Center for Analytical Sciences, State Key Laboratory of Medicinal Chemical Biology, Tianjin Key Laboratory of Biosensing and Molecular Recognition, Nankai University, Tianjin, 300071, China

4 Central Laboratory, Xuanwu Hospital Capital Medical University, Beijing Municipal Geriatric Medical Research Center, Beijing 100053, China

5 Department of Pathology, School of Medicine Nankai University, Tianjin, 300071, China

6 National Medical Center for Neurological Diseases, Beijing 100053, China

7 Clinical Research Center for Epilepsy Capital Medical University, Beijing 100053, China

* Correspondence author:

Guoguang Zhao

Email: ggzhao@vip.sina.com

Yuhao Li

Email: liyuhao@xwhosp.org

Wenyou Li

E-mail: wyli@nankai.edu.cn

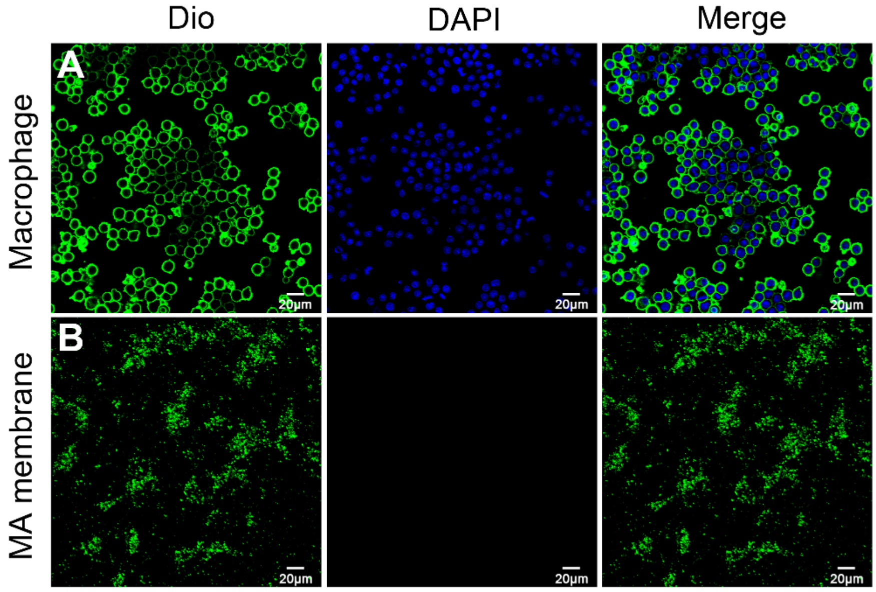

Figure S1. DiO fluorescence staining of the RAW264.7 macrophage membrane. (A) DiO fluorescence staining image of the RAW264.7 cell membrane. (B) Fluorescence images of green cell membrane fragments after cell membrane extraction. Scale bar in (A) and (B), 20 μm.

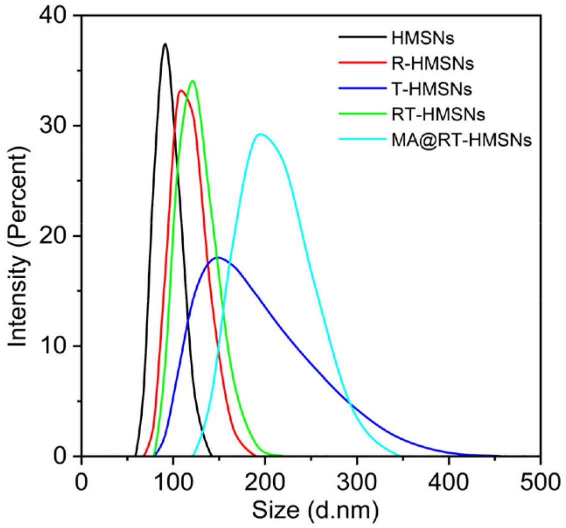

Figure S2. Hydrated particle sizes of HMSNs, R-HMSNs, T-HMSNs, RT-HMSNs and MA@RT-HMSNs by DLS.

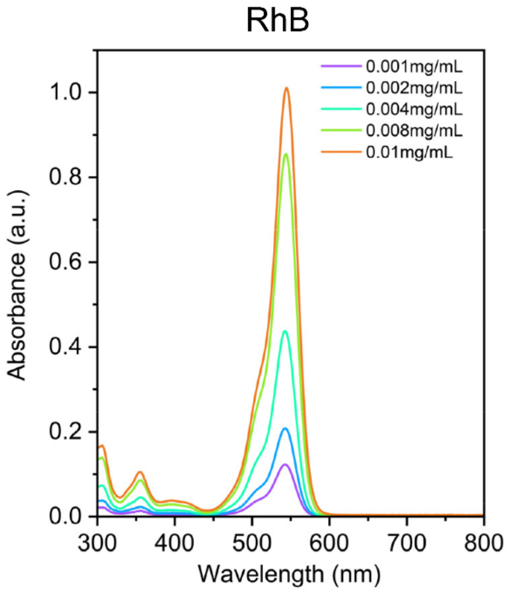

Figure S3. UV–vis absorption spectra of RhB at different concentrations.

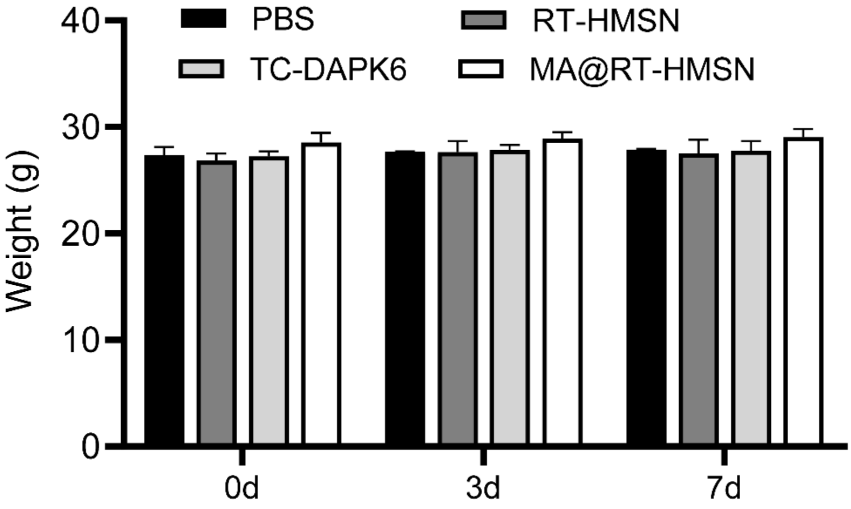

Figure S4. Body weight analysis of the mice at 0, 3, and 7 days following PBS, RT-HMSN, TC-DAPK6 or MA@RT-HMSN injection.

No significant difference was found among the four groups (n = 3 in each group).

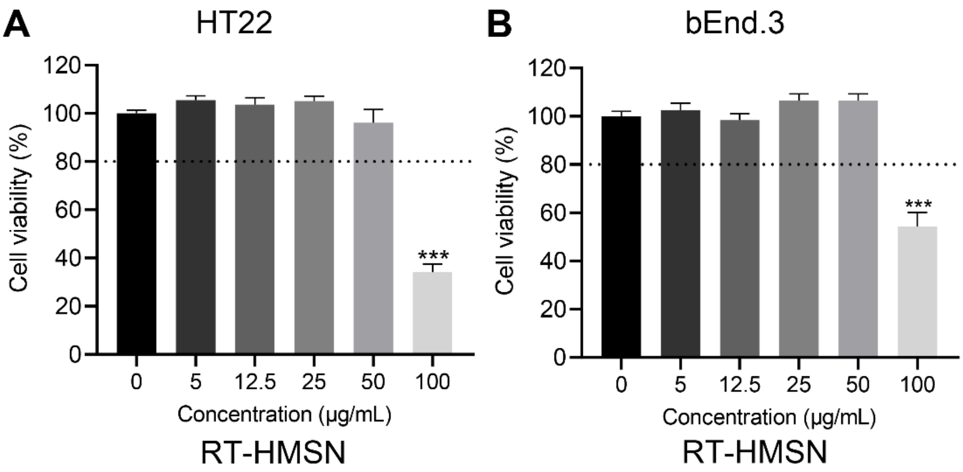

Figure S5. Cell viability of (A) HT22 and (B) bEnd.3 cells incubated with RT-HMSNs at various concentrations (0, 5, 12.5, 25, 50, 100 μg/mL) for 24 h (ANOVA; *** P < 0.001; n = 6 at each concentration).

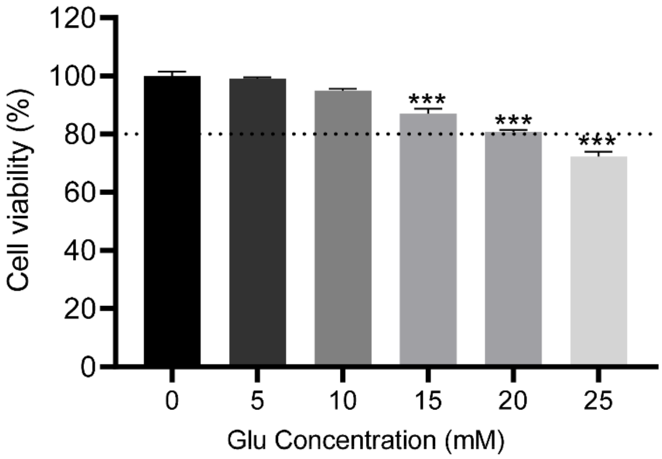

Figure S6. Cell viability of HT22 cells treated with various concentrations of glutamate (0, 5, 10, 15, 20 and 25 mM) for 24 h (ANOVA; *** P < 0.001; n = 6 at each concentration).

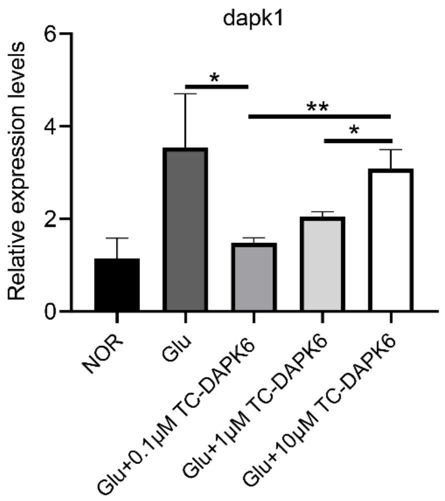

Figure S7. qRT–PCR analysis of the mRNA expression of *dapk1* in HT22 cells treated with glutamate and different concentrations of TC-DAPK6 (ANOVA, $*P < 0.05$, $**P < 0.01$; $n = 4$ in each group). NOR, normal control.

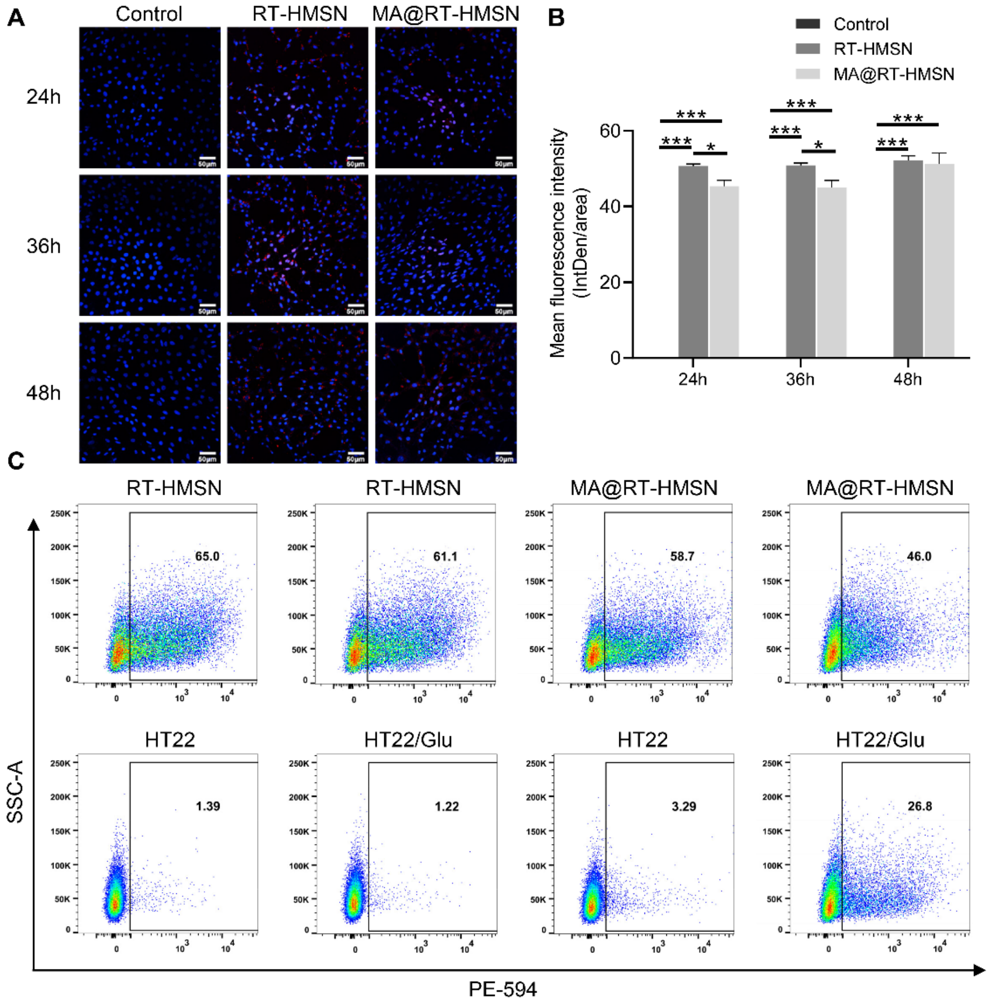

Figure S8. *In vitro* fluorescence imaging and BBB penetration of MA@RT-HMSNs. (A) Fluorescence images of HT22 cells following RT-HMSNs or MA@RT-HMSNs exposure for 24, 36 or 48 h. (B) Quantitative analysis of the mean fluorescence intensity (ANOVA; *P < 0.05, ***P < 0.001). (C) Flow cytometry analysis of the cellular uptake of RT-HMSNs and MA@RT-HMSNs in bEnd.3 cells (upper four panels in C) and in HT22 cells (lower four panels in C) of the BBB model.

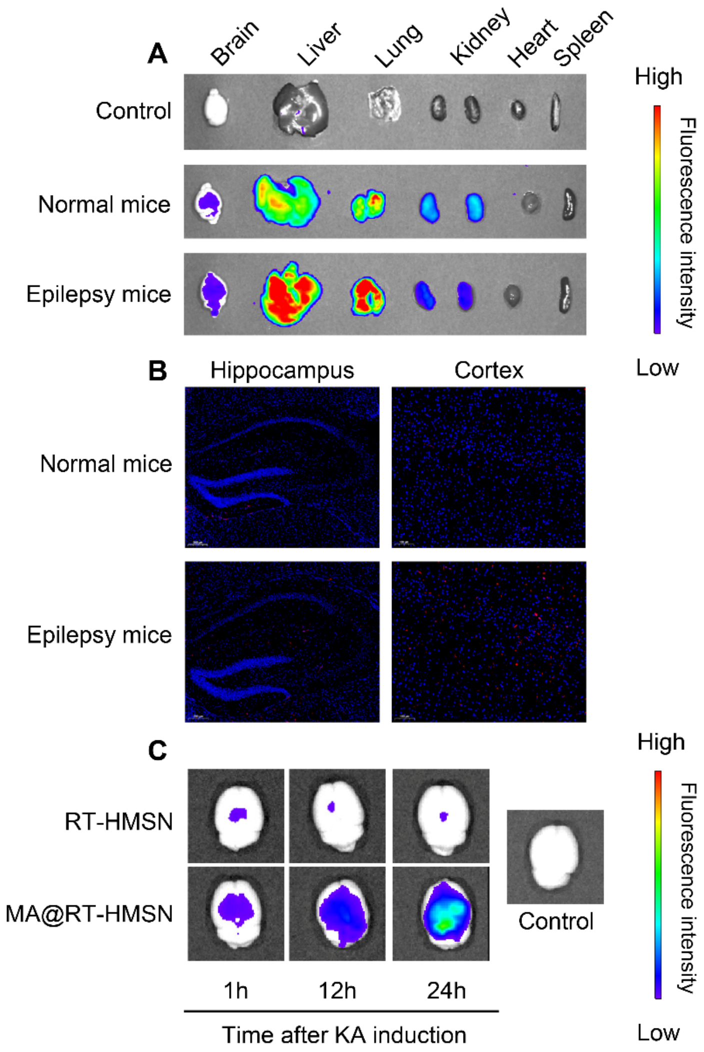

Figure S9. *Ex vivo* imaging of MA@RT-HMSNs in the brain with RhB as a fluorescent probe. (A) *Ex vivo* IVIS imaging of the distribution of MA@RT-HMSNs in the brain and major organs (liver, lung, kidney, heart and spleen) after tail vein injection in

uninjected (control), normal and epilepsy model mice. (B) Microscopic distribution of MA@RT-HMSNs in the hippocampus and cortex of normal and epileptic mouse brains. (C) *Ex vivo* imaging of RT-HMSNs and MA@RT-HMSNs in epileptic mouse brains at different time points (1, 12, and 24 h).

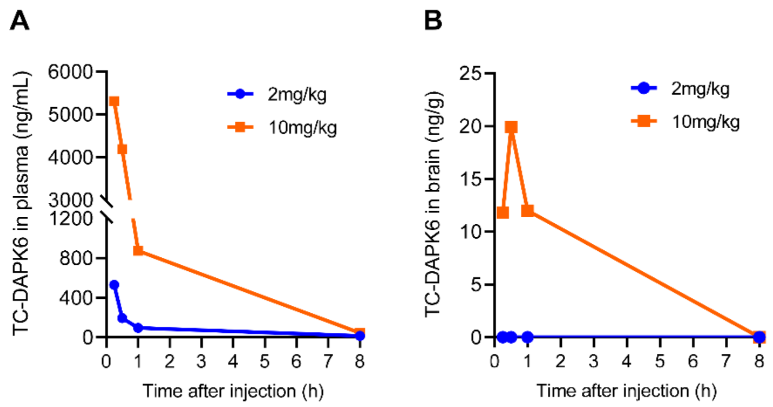

Figure S10. Concentrations of TC-DAPK6 in the (A) plasma and (B) brain after intraperitoneal injection in mice.

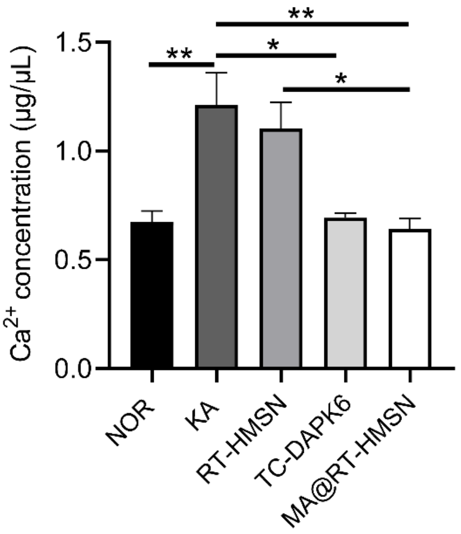

Figure S11. Determination of hippocampal Ca^{2+} concentrations in the NOR, KA, RT-HMSN, TC-DAPK6 and MA@RT-HMSN groups (ANOVA, **$P < 0.01$; $n = 6$ in each group). NOR, normal control.

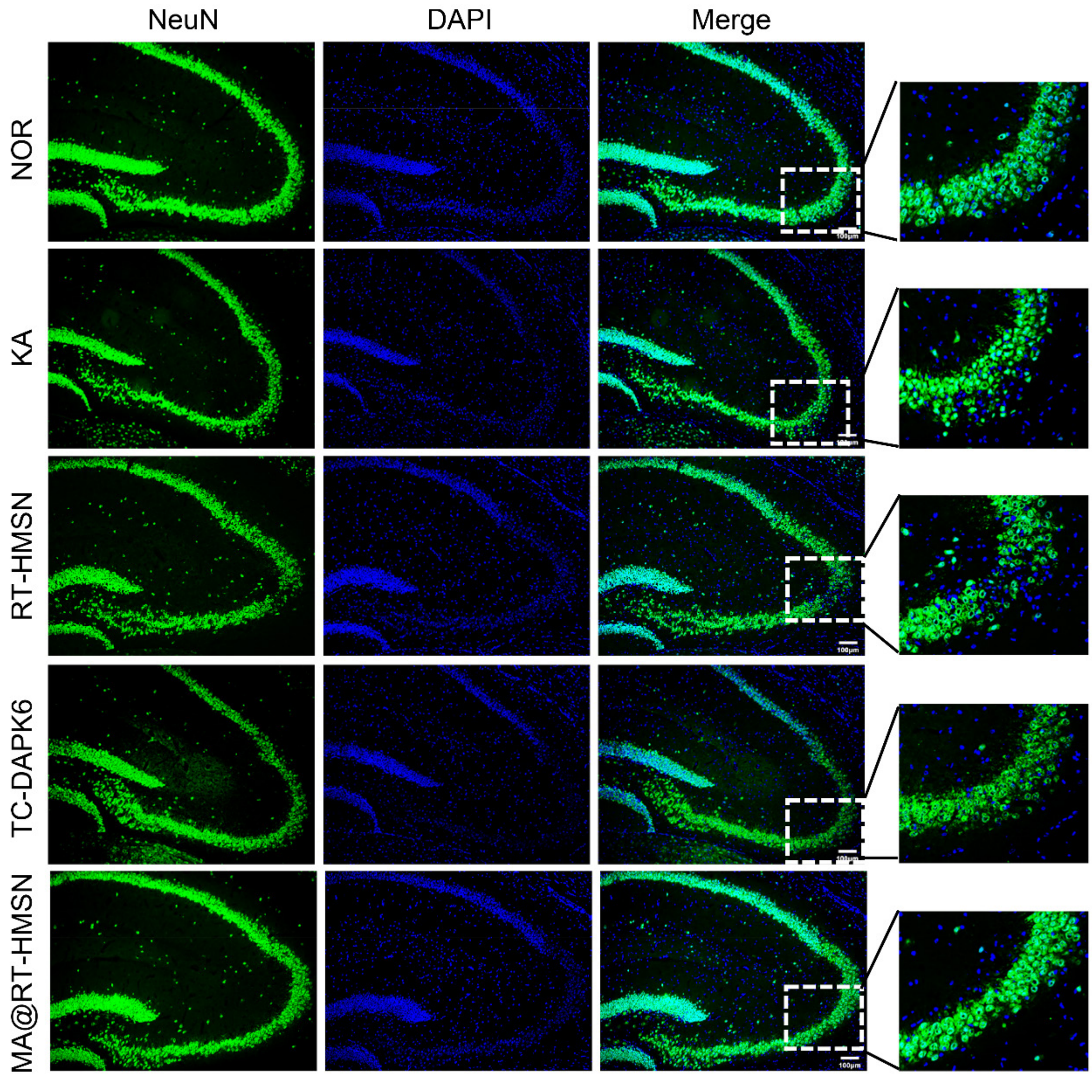

Figure S12. Images of NeuN immunostaining in the NOR, KA, RT-HMSN, TC-DAPK6 and MA@RT-HMSN groups. The lesion sites are indicated by the dotted rectangles with magnified images on the right. Scale bar, 100 μm. NOR, normal control.

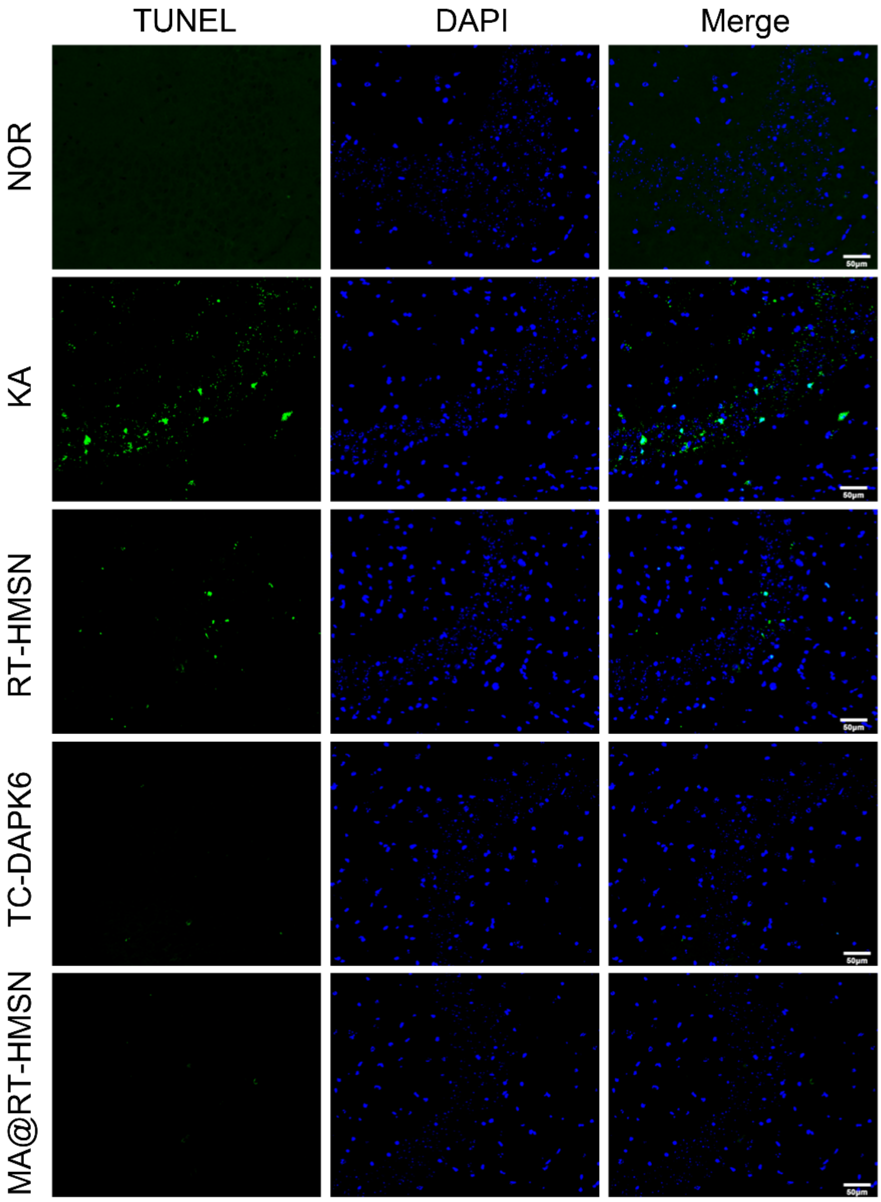

Figure S13. TUNEL staining of apoptotic neurons in the NOR, KA, RT-HMSN, TC-DAPK6 and MA@RT-HMSN groups. Scale bar, 50 μm. NOR, normal control.

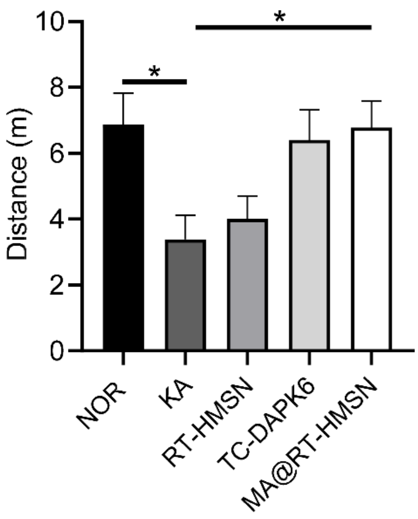

Figure S14. Statistical analysis of the distance on rotarods of the mice in the NOR, KA, RT-HMSN, TC-DAPK6 and MA@RT-HMSN groups (ANOVA, *P < 0.05; n = 6 in each group). NOR, normal control.

Table S1. Primer sequences for qRT–PCR.

Genes	Primer	Sequence (5‘-3’)
c-fos	Forwards	GAATCCGAAGGGAACGGAATAA
	Reverse	GCAACGCAGACTTCTCATCT
dapk1	Forwards	CCATTCGGGATCAGGGAAATC
	Reverse	GGTGGAGTTGGTAGAGGATAGT
Caspase3	Forward	GGAAAGCCGAAACTCTTCATCATTC
	Reverse	GCAAGCCATCTCCTCATCAGTC
il-1β	Forwards	AAAGCTCTCCACCTCAATGG
	Reverse	CCCAAGGCCACAGGTATTT
tnf-α	Forwards	TGTCTACTCCCAGGTTCTCTT
	Reverse	GCAGAGAGGAGGTTGACTTTC
il-6	Forwards	CTTCCATCCAGTTGCCTTCT
	Reverse	CTCCGACTTGTGAAGTGGTATAG
β-actin	Forwards	CTACCTCATGAAGATCCTGACC
	Reverse	CACAGCTTCTCTTTGATGTCAC
